# The living Barents Sea response to peak-warming and subsequent cooling

**DOI:** 10.1038/s41598-025-96964-x

**Published:** 2025-04-15

**Authors:** E. Eriksen, B. Husson, G. Skaret, R. B. Ingvaldsen, P. Dalpadado, E. Johannesen, L. L. Jørgensen, B. Bogstad, A. V. Dolgov, D. V. Prozorkevich, T. A. Prokhorova, A. A. Russkikh, N. A. Strelkova, A. G. Trofimov, I. P. Prokopchuk, A. A. Filin

**Affiliations:** 1 Institute of Marine Research, Bergen, Norway; 2 Polar Branch of Russian Federal Research Institute of Fisheries and Oceanography (“PINRO” Named After N.M. Knipovich), Murmansk, Russia

**Keywords:** Warming, Climate change, Sub-Arctic, Ecosystem components, Redistribution, Biomass, Ecology, Climate-change ecology

## Abstract

The Arctic warms nearly four times faster than the global average, with maximum warming in the Barents Sea. Concurrently, changes in species distribution in this productive and highly exploited sub-Arctic hotspot has been found. However, studies so far have mostly focused on the effect of gradual warming on single species or trophic groups. We assess changes in zooplankton, fish and zoobenthos assemblages (130 species in 23 groups) and found heterogeneous response to ongoing warming. Temporally constrained cluster analysis showed that the warming was not continuous over the study period 2005–2022 but occurred in three phases: an initial period (2005–2011) cooler than the average for the whole study period, followed by a very warm period (2012–2016) and finally a cooler period again (2017–2022). The biotic response was greatest in the areas of largest oceanographic changes: in the northwest, the biomass of biota increased in most groups, including Arctic fish species, whereas in the southeast, the biomass of several fish species declined, while that of jellyfish and invasive snow crab increased. New knowledge is useful for generating scenarios for ecosystem responses to climate change.

## Introduction

Due to global warming, the Atlantic and Arctic oceans are undergoing shifts in currents and temperature patterns, along with increased melting of ice^1^. The Barents Sea (BS) is a transition zone between the Northeast Atlantic and Arctic Oceans and is strongly influenced by large scale atmospheric and oceanic processes^2^. The oceanographic conditions in the BS are reflected in the biota: the southern ice-free BS is dominated by boreal species, whereas the northern seasonally ice-covered BS is dominated by cold water associated species^3–5^. However, due to Atlantic advection and seasonal migrations, boreal species may also occur in the northern BS during summer-autumn^6^.

In the BS, warming above the global mean temperature has been driven by a combination of warmer inflows from the south and regional processes enhancing the warming (reviewed by Ingvaldsen^7^). Along with warming, the region dominated by Atlantic waters strongly expanded north-eastwards^8^ thereby causing a substantial reduction in sea ice area (e.g. Ref^9^ and increase in air temperatures (e.g. Ref^10^). However, the warming in the region is not linear but observed as pulse-like events on top of a general increase in temperature, with the most recent warming event peaking in 2015–2016^7^.

The response in the living parts of the ecosystem to pulses of warming and cooling is likely dependent on life history traits of marine organisms such as mobility and generation time, as well as trophic position^11^. Responses might also be indirect through ecological interactions^12,13^, behavioural or demographic, reversible or more permanent^14–17^. There is a lack of knowledge on how different living communities in the BS ecosystem respond to periodical warming and cooling on top of an unprecedented warming trend in the ecosystem, and also on how the responses might vary spatially. Many studies have focused on the unprecedented rise in temperature since the early 2000s and up to the 2015–2016 warming event, which has been associated with large fluctuations in species abundance and shifts in distribution (reviewed by Gerland^18^). There is, however, a lack of knowledge about the response of the living ecosystem components in the BS since then.

**Fig. 1:**
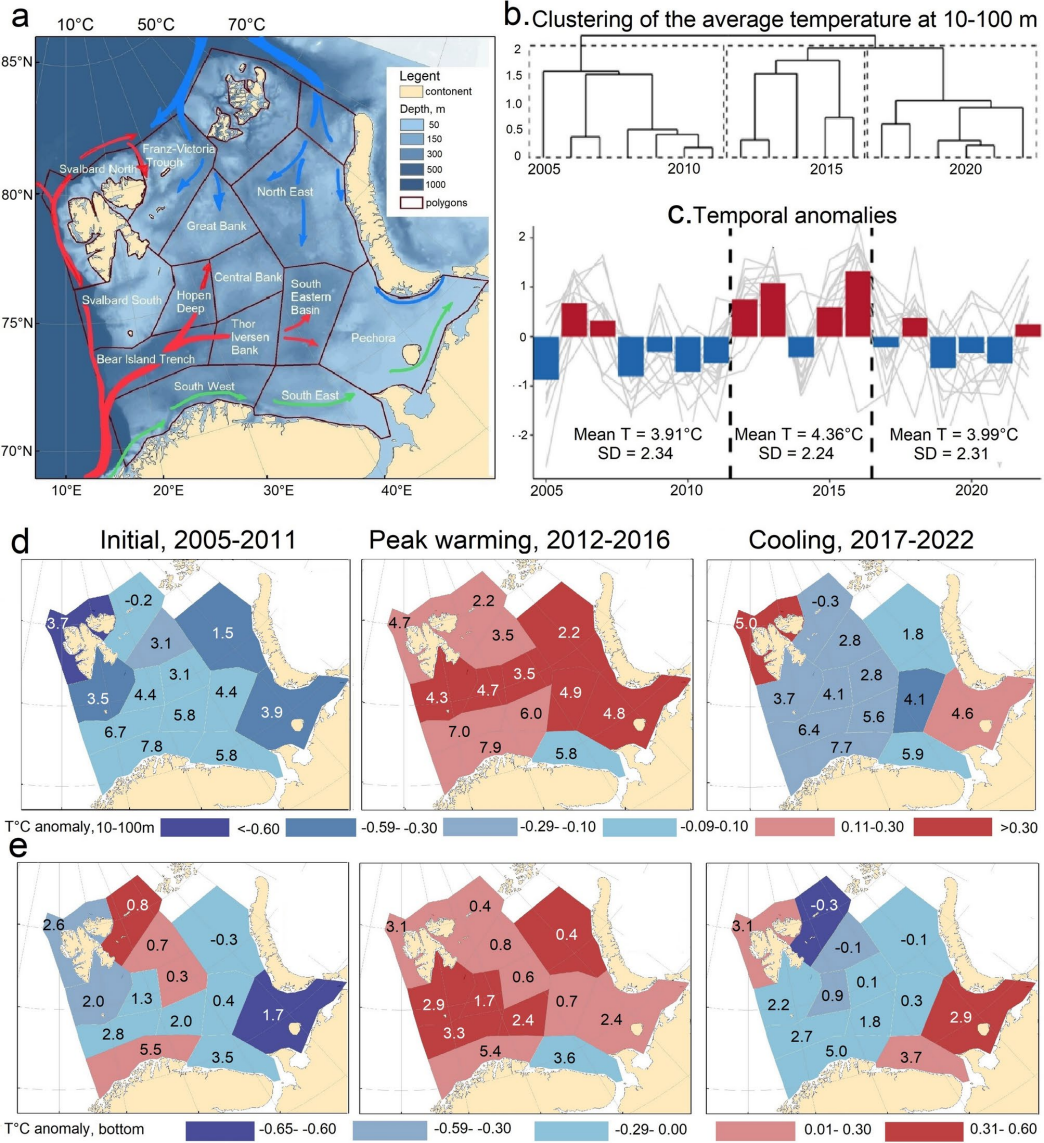
(**a**) The Barents Sea with main ocean currents: Atlantic inflow in red, Arctic in blue and Coastal in green (higher resolution map shown in supplementary Figure S2-0), (**b**) Three time periods characterized by different thermal conditions identified through temporally constrained hierarchical clustering of the average temperature at 10–100 m. (**c**) time series of temporal anomalies for 2005–2022. The bars show average late summer temperature anomalies for all polygons combined while the gray lines show late summer temperature for each polygon. The three identified time periods are separated by dashed vertical lines and averaged temperature for each period with associated standard deviation is given. (**d**) Colors show periodical temperature anomalies (°C) in relation to average temperature in the polygon for the whole study period 2005–2022, while numbers show average temperature at 10–100 m and (**e**) at the bottom per polygon and period.

In this integrative study, we investigated how abundance and distribution of the living ecosystem components in the BS respond to periodical variation in climatic conditions and whether a possible response is (i) greatest in areas with largest change in oceanographical conditions (ii) dependent upon the organisms’ trophic and functional position in the ecosystem and (iii) causing change in the BS elasticity (ability of the system to change under the influence of external forces and to return to its initial state when these forces cease). We explored data from the comprehensive Barents Sea Ecosystem Surveys (BESS^19^) in the summer-autumn period (August–September) and documented the temporal (2005–2022) and spatial (Fig. 1a) changes across 23 abiotic/biotic groups combined into 6 main assemblages: (i) oceanographic and sea ice conditions, (ii) zooplankton, (iii) 0-group fish (age 0), (iv) pelagic fish and (v) demersal fish, and (vi) zoobenthos. A total of 299 time series were calculated, representing 4 abiotic and 19 biotic groups distributed in 13 spatial polygons (Table 1, Fig. 1). These time series were used to assess the response of the living BS to climate change (Table 1).

**Table 1:** Grouping of the Barents Sea ecosystem components into assemblages and abiotic and biotic groups. The number of time series within each group is given in brackets.

Environment (4 groups)		
Environmental conditions	Oceanographic conditions (2)	1. Water temperature at 10–100 m and 2. Bottom water temperature
	Ice condition (2)	1. Number of days of ice and 2. Open water area
The living BS (19 groups)		
Plankton	Mesozooplankton (1)	
	Macrozooplankton (3)	1. Jellyfish, 2. Krill, and 3. Amphipods
0-group fish	Pelagically distributed juvenile fish (4)	1. 0-group boreal commercial fish, dominated by cod (8 species), 2. 0-group capelin, 3. 0-group polar cod, 4. 0-group herring
Pelagic fish	Pelagic fish, age 1 and older (4)	1. Capelin, 2. Young herring, 3. Polar cod and 4. blue whiting
Demersal fish	Commercial demersal fish, age 1 and older (1)	Commercial demersal species, dominated by cod and haddock (12 species)
	Other large demersal fish (1)	Large demersal fish, dominated by wolffishes (8 species)
	Small arctic demersal fish (1)	Small arctic demersal fish dominated by bigeye sculpin (10 species)
	Small boreal demersal fish (1)	Smal boreal demersal fish, dominated by Norway pout (10 species)
Benthos	Arctic zoobenthos (1)	Dominated by Echinoderms (79 species)
	Boreal zoobenthos (1)	Dominated by Sponges (44 species)
	Commercial zoobenthos (1)	Red king crab, snow crab, northern shrimps

## Periodic warming and environmental variability

Temporally constrained cluster analysis^20^ of the 10–100 m mean temperature (Fig. 1b) showed that the warming was not continuous over the study period, and three periods with different thermal conditions across the sub-areas in the BS were identified (Fig. 1c–e). These included an initial period (2005–2011) cooler than the average for the whole study period, followed by a very warm period (2012–2016) and finally a cooler period again (2017–2022). The three periods are hereafter called the initial period (recognizing that it is substantially warmer than the 1980s and 1990s), peak warming, and cooling, respectively.

Temperatures at 10–100 m during the peak warming period were ca. 0.4 °C higher than during the other two periods (Fig. 1c). The peak warming period was characterized by increased temperatures in all sub-areas except the South-East polygon (Fig. 1d). The most significant warming (0.9–1.0 °C increase) occured in the Svalbard North and the Pechora sub-areas (Fig. 1d). During the cooling period, temperature in most polygons returned to similar levels or even lower compared to the initial period, while temperatures in the Svalbard North and Pechora sub-areas were similar or became even higher compared to the peak warming period (Fig. 1d).

Bottom temperature anomalies showed a similar periodic spatial pattern as temperatures at 10–100 m, with some exceptions (Fig. 1e, supplementary Fig. S1-1), like the Fr. Victoria Trough region that had lower temperatures during the cooling period than in the two former periods, and the highest temperatures in the initial period (Fig. 1e).

## The response of the biota to environmental change

### Main changes in biota over the three periods

The biotic groups (Table 1) that showed largest changes over the three periods are described below.

**Fig. 2:**
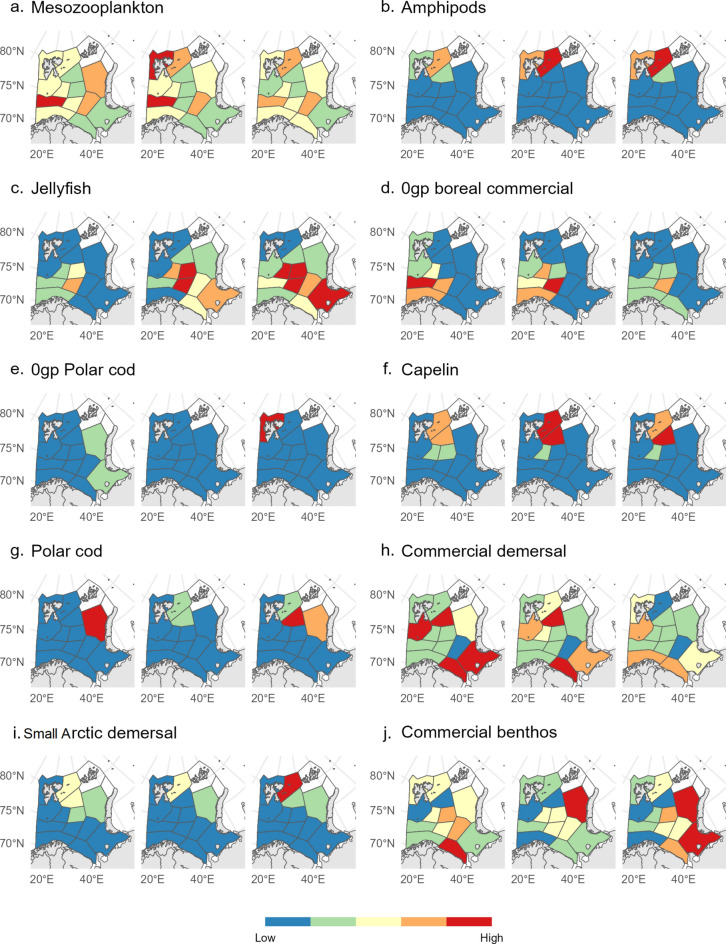
Mean biomass of selected biotic groups shown by sub-area and period. Plots of the other biotic groups (Table 1) are presented in supplementary Fig. S1-3–7. The biomass range here is shown in a simplified way, from low (blue) to high (red), whereas the mean biomass values and ranges for all biotic groups are shown in supplementary Fig. S1-3–7.

**Fig. 3:**
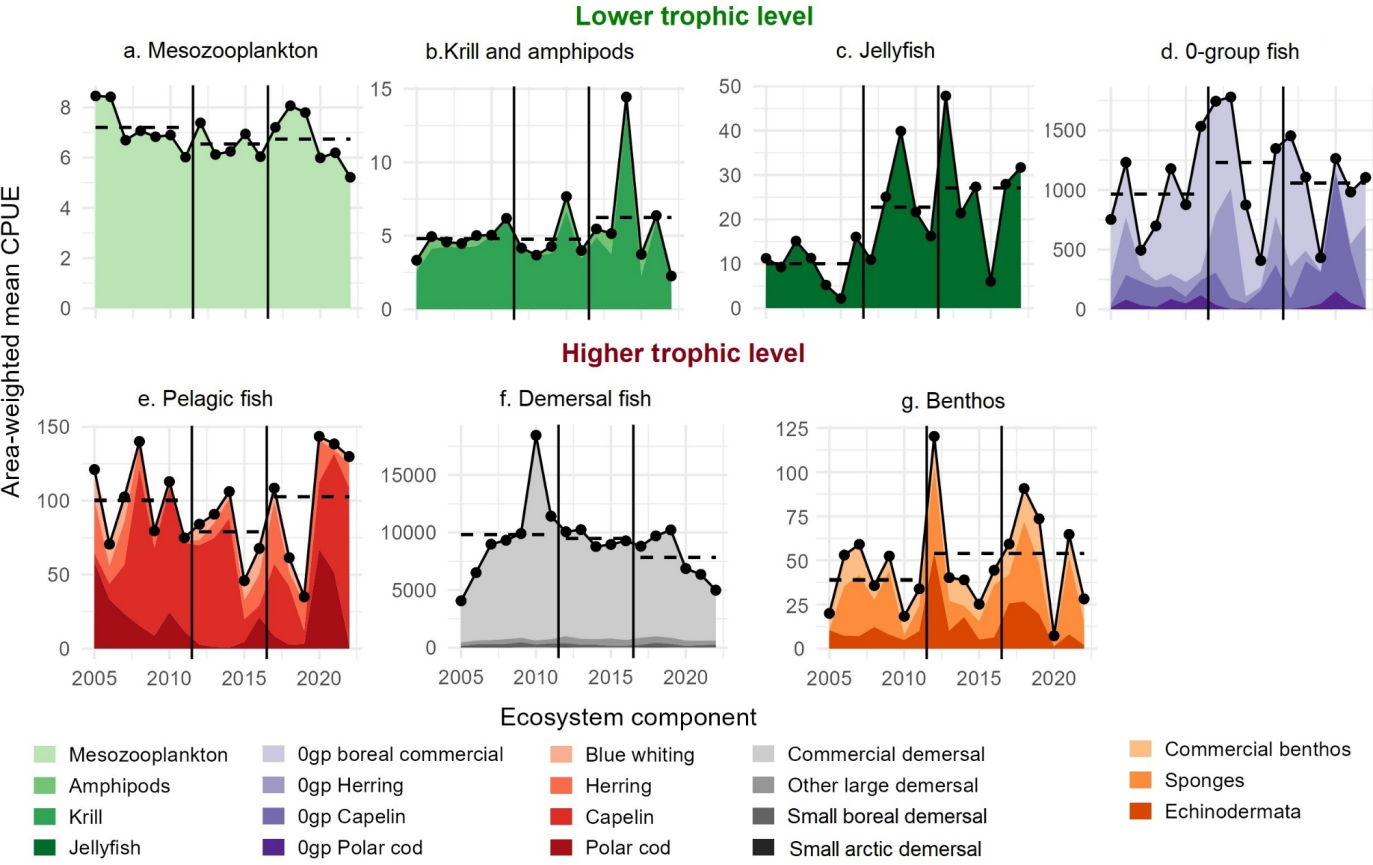
Area-weighted mean catches standardized by effort (CPUE) for different assemblages. The observed values for different assemblages are displayed, including those for mesozooplankton (g dry weight/m^2^), while krill, amphipods, jellyfish, fish and benthos (kg/km^2^). The three identified time periods are separated by black vertical lines. Area-weighted mean catches standardized by effort for different species are also presented in supplementary Fig. S1-9. The dots represent the cumulative mean value for the biotic group.

Both jellyfish and 0-group of boreal commercial fish significantly increased their distribution range over the three periods. Distribution of jellyfish dominated by *Cyanea capillata* expanded from mainly the central BS in the initial period to all sub-areas except for the two northernmost in the cooling period (Fig. 2c). Also, biomass of jellyfish increased in almost all sub-areas from the initial to the peak warming and continued to increase in the cooling period (Fig. 3, supplementary Fig. S1-2h). The increase in jellyfish biomass was especially pronounced in the Pechora sub-area. Also 0-group of boreal commercial fish increased in biomass in the east (Southeastern Basin and the South-East sub-areas) during peak warming (Fig. 2d). The biomass remained high in these sub-areas during the cooling.

The distribution area of capelin, 0-group/adult polar cod, and small arctic fish decreased strongly. Capelin disappeared from the two southernmost sub-areas of its distribution range (Thor Iversen Bank and South Eastern Basin) from the initial to the cooling period (Fig. 2f and supplementary Fig. S1-2o). Also, adult polar cod disappeared from the sub-areas in its southern distribution range (Central Bank, Southeastern Basin and Pechora) from the initial to the peak warming period and did not reappear in the cooling period (supplementary Fig. S1-2p). 0-group polar cod disappeared from the eastern tarts of its distribution area (North East and Pechora polygons) from the initial to the cooling period, and during the cooling period they were only found in the sub-areas to the north-west (Fig. 2e, g). The small arctic fishes almost disappeared from the three southernmost sub-areas of its distribution range (Central Bank, Southeastern Basin, and Pechora sub-areas) from the initial to the peak warming period and did not return during the cooling period (Fig. 2i, supplementary Fig. S1-2t).

### Biotic response in the Svalbard North and Pechora regions

Both Svalbard North and Pechora sub-areas did not cool in the last period but rather continued to warm. Both showed large environmental change over the study period (Fig. 1). However, these sub-areas were very different in terms of inter-annual dynamics in biomass of the biotic groups, with Svalbard North showing much higher cumulative interannual variability than Pechora (supplementary Fig. S1-8). Both sub-areas had substantial change in the biota.

In the Svalbard North sub-area, biomasses of many biotic groups increased across the three periods. Biomass of krill and 0-group capelin which are all advected from the south into the sub-area and 0-group polar cod, showed clear increases across all periods in this polygon (Fig. 2 and supplementary Fig. S1-3, 4). Biomass of amphipods, jellyfish, capelin, commercial demersal fish, and other large demersal fish (Fig. 2 and supplementary Fig. S1-6b) also increased, but less than the above-mentioned groups. Biomass of polar cod and 0-group boreal commercial species on the other hand decreased throughout the study period, while other groups varied more between periods (Fig. 2).

In the Pechora sub-area, biomass of jellyfish and commercial benthos (snow crab) strongly increased, while biomasses of both commercial demersal fishes and small arctic fishes decreased. Biomass of 0-group polar cod also clearly decreased, while capelin and polar cod biomass decreased to almost zero.

### Biotic response in relation to trophic and functional position

The lower trophic level represented by zooplankton (meso- and macroplankton) and 0-group fish, generally expended their distribution from the first to the second period, except for jellyfish, which continued to increase their distribution also in the last period (Fig. 2a–c and supplementary Fig. S1-3, 4). The biomass of the low trophic organisms showed no clear relation to the temperature conditions (Fig. 3).

For organisms further up the food chain, including fish species with longer life spans, biomass and distribution were highly variable between species, areas and periods, with no clear relationship to temperature conditions, except for the arctic species (see above and Fig. 2g, i and 3).

The most significant changes in the commercial benthos group were related to a substantial increase in biomass of the cold-water snow crab in the eastern part of BS (Fig. 2j), despite the high temperature in the southeast (1e). Other benthic groups showed no clear relationship with changed temperature conditions (Fig. 3).

A full, extensive documentation of the state and changes in the living BS from zooplankton to fish and benthos communities in addition to oceanographic conditions are provided in supplementary materials S1 (maps), S2-1–14 (time series) and S3 (integrated analyses).

## Discussion

The present comprehensive study includes the most recent cooling but still warm period in the Barents Sea, thereby expanding on previous integrated studies of the Barents Sea^21–24^. This enables us to investigate the response and resilience of the system to cooling after peak warm conditions.

The biomass of jellyfish in the BS is dominated by lion’s mane jellyfish (*Cyanea capillata*^25^). A study of global jellyfish rise revealed that they exhibited decadal oscillations, with two previous peaks globally occurring in the 1971–1985 and 1993–2004 periods^26^. Previously (1993–2013) in the BS, high jellyfish biomasses occurred in two peaks associated with high temperature, namely in 2001–2003 and 2013^27^. In this study we documented four additional peaks (2014, 2017, 2019 and 2022, Fig. 3) thus occurring with a higher frequency than previously observed. Jellyfish limiting the habitat of pelagically distributed fishes (juvenile or adults) has been observed in many regions, but has not in the BS^27^. With rising temperatures, a declining fish biomass in the Pechora sub-area was observed. The reasoning behind these changes should be explored further, also in a wider ecosystem context.

Our results indicate that increasing temperatures negatively influenced the biomass of arctic fish species in the south. The polar cod, which is a mid-trophic keystone species in the arctic part of the Barents Sea, strongly declined in abundance in the south (South Eastern Basin and Central Bank) and almost vanished from Pechora during summer-early autumn. Earlier studies have linked the decline of polar cod in the south to spawning collapse caused by the retreating winter ice^28^. Our study supports this and indicates that this trend has continued despite the recent cooling. The shift we observe in the distribution of 0-group polar cod from south-east to north-west also indicates that spawning in recent times mainly happens in the north. In the BS, the very low abundance of polar cod in 2018–2019, and the simultaneous occurrence of a very strong year class and high abundance in 2020, suggests a connectivity between the BS and the Kara Sea polar cod stocks. Ingvaldsen^7^ mentioned that sea ice import from the Kara Sea have become more important for the BS in recent years. Thus, the BS connectivity with the Kara Sea likely dampens consequences of the diminishing of Arctic habitat and biota by serving as a refill of ice and polar cod following the sea ice drift, thereby supplementing the BS population.

Similar to polar cod, small arctic demersal fishes have almost disappeared from their southernmost distribution sub-areas (the Central Bank, the South Eastern Basin and Pechora, Fig. 2i) during the peak warming period and did not return during the cooling period. Reduced appropriate thermal habitat may explain the lack of return, but probably other factors such as food availability and low mobility may have contributed. A similar mechanism may explain the reduction of capelin in these sub-areas. Interestingly, our updated biomass time series of the small arctic demersal fishes showed that they increased in the northern sub-areas (Franz Victoria Trough and Great Bank) during the cooling period, while previous studies have predicted displacement of these by boreal fishes^21^. The increasing ice cover and reduced predation from the declining cod population might explain this return.

A considerable number of recent studies have focused on the redistribution of marine species due to climate change, but few examine the species ability to return to previous states once the driving forces are relaxed. This study highlights this resilience using small Arctic species in the northern BS as an example. Burrows^29^ suggest that marine species redistribute by following their preferred temperatures. However, it is unclear to what degree small Arctic demersal fish can migrate long distances in response to rapid their habitat changes. Their response to future warming remains uncertain because the underlying mechanisms of the redistribution, return and connectivity with other areas are not fully understood.

The harvested fish populations in general showed notable dynamics that could not readily be linked to environmental change at sub-area scale over the three periods identified here. The decline in the biomass of commercial boreal species, dominated by cod and haddock, was not in line with expectations of increase due to warming, since the biomass showed a continuous decline during both during peak warming and in the last period. As commercial stocks have declined over the last decade, other large demersal species have increased (supplementary Fig. S1-6b).

It is known that the fish populations such as herring, capelin, cod and haddock have strong interannual variability in recruitment that contribute to population dynamics^12,31,32^. We found that the distribution of 0-group boreal fish increased over the study period but without a significant increase in biomass. Local growth conditions and species interactions^33^, which were not investigated here, may be important in determining the lack of 0-group biomass increase, despite increase in distribution.

The strong increase in snow crab was the most significant change observed for the commercial benthos. This species is a newly occuring species in the BS^34^. Despite being a cold-water species, it did not seem that warming of the Pechora (from 1.7 to 2.9 °C) or North-East (from − 0.3 °C to 0.4 °C) sub-areas limited the biomass of the snow crab. An earlier study indicated that higher temperatures may slow the westward expansion of the snow crab^35^. Our study showed that several sub-areas with lower temperatures were more suitable thermal habitat than in the eastern sub-areas.

**Fig. 4:**
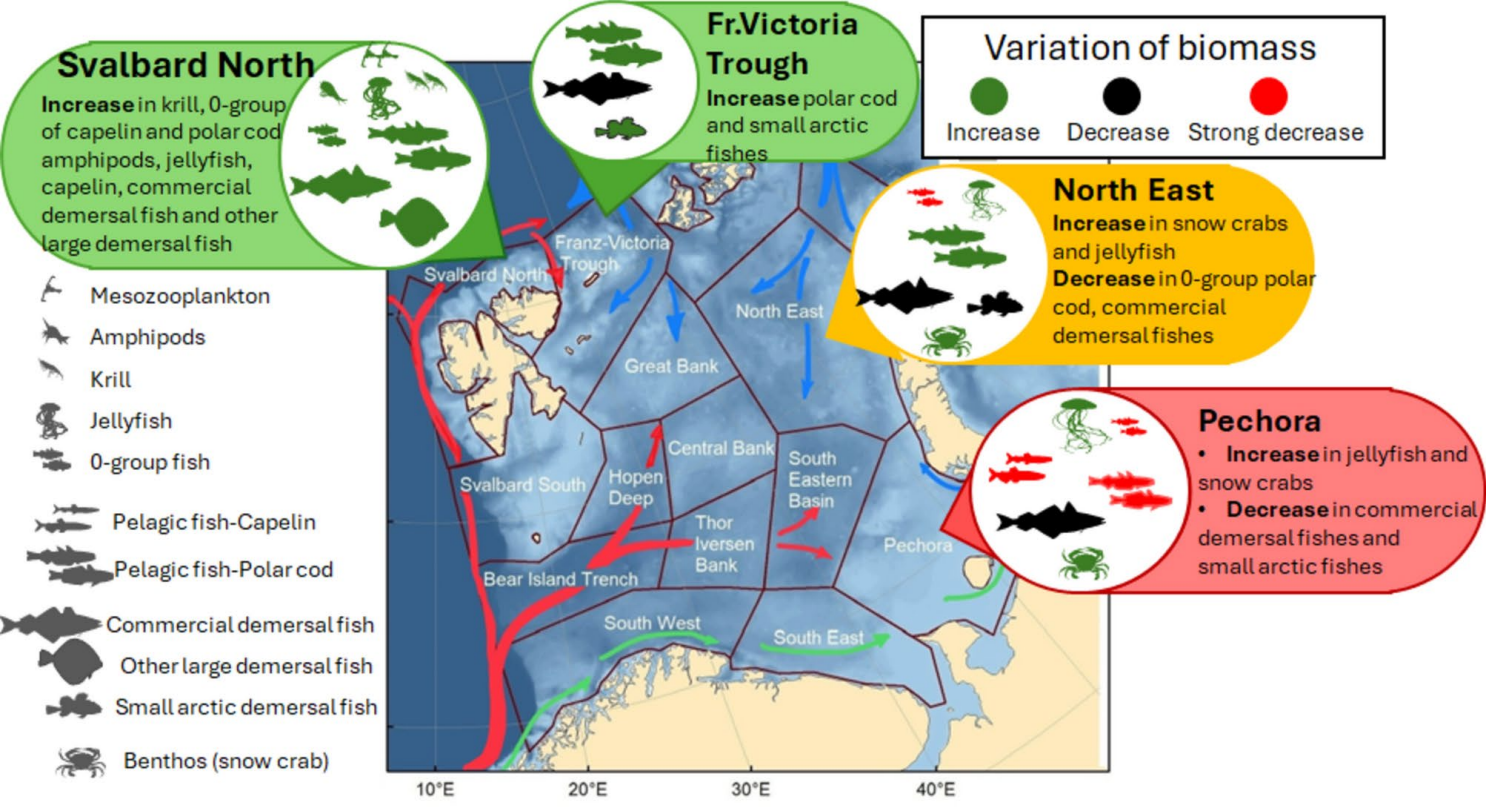
The greatest changes in the living BS (in coloured boxes, from green showing changes we judge to be positive whereas red showing more negative changes) since 2005. Increase in biota colored by green, decrease in black and disappearance in red.

The greatest biological changes were observed in the areas with strongest oceanographic changes (Fig. 1d,e), in the northwestern and southeastern BS (Fig. 4). The Svalbard North and Pechora sub-areas experienced an exceptional warming throughout the water column in the peak warming period, and temperatures remained high or even higher (near bottom, Pechora sub-area) in the cooling period. The shift of polar cod into the northern BS during the cooling period indicated suitable feeding and spawning conditions there. Although the northern BS had high inter-annual variability, the food web here showed a high degree of adaptability to climate change with an increase in the boreal community during the peak warming period and an increase in the arctic community with ice retreat and cooling.

The southeastern BS experienced less cumulative environmental and biological change, with less variation from year to year compared to the northwest, but overall, the ecosystem changed considerable over the study period. The shallow southeast is mostly influenced by coastal waters and to a lesser extent Atlantic saline waters^32^.The generally low biomass of both mesozooplankton and fish in this region has declined further during the study period, and the substantial increase in jellyfish and snow crab appears to have changed the ecosystem there to a new state. Both jellyfish and snow crab are insufficient food for fish, but their diet may partly overlap that of fish, meaning more compitition.

## Conclusion

Our results show that the ongoing warming of the BS is not linear but rather occurs in different phases and exhibits notable regional variation. The BS ecosystem showed a heterogeneous response to ongoing warming in term of different phases, areas and biota and the ability of living organisms to return to initial state when driving forces are weakened.

The biotic response were greatest in the areas of largest oceanographic changes: the Svalbard North biota went through changes in the form of an increase in biomass of many ecosystem components, whereas living Pechora underwent biomass decrease of several fish species and an increase in jellyfish and invasive snow crab.

We found both previously described biotic responses to climate change, such as the redistribution of boreal fish species as Atlantic water expands, and unreported responses, such as the reappearance of small Arctic demersal species as Arctic water returns. The northern limit of the cod distribution may have followed the expansion and retreat of Atlantic waters in the northern BS, but the main reason is likely increase and decline of the stock. In addition to the previously described decadal “peaks” of jellyfish, our data indicated several interannual “peaks” as well as a strong increase in jellyfish in the Pechora sub-area.

A key issue for the ecosystem response to climate change appears to be increases in species that have the potential to temporarily or permanently alter the food webs. Such changes are challenging to assess or predict without a comprehensive monitoring of the ecosystem.

## Methods

### Data collection

Data on species biomasses and environmental conditions were collected annually from 2005 to 2022 during the joint Norwegian-Russian ecosystem survey in August–September^19^. The survey employed on average 278 fixed stations across the Barents Sea each year, with approximately 35 nm between stations, except for the western and northern slopes of Svalbard/Spitsbergen where the distance between stations was shorter. At each station, the vessel sampled most compartments of the ecosystem using a pelagic trawl (Harstad trawl) for capturing macroplankton (krill, amphipods and jellyfish), 0-group and older fish, hauled over 3 × 20 meter at different depth in upper 60 m^19^, WP2 and Juday nets (180 µm mesh size) for sampling mesozooplankton covering entire water column^36^, Conductivity-Temperature-Depth (CTD) profiles, and a bottom trawl (Campelen 1800, 22 mm mesh size at the cod-end, trawled at 3 knots for 15–30 min) for capturing megabenthos and demersal fish. Adult pelagic species abundances were estimated from acoustic registration all along the survey tracks using EK-type echosounders, and NASC was then averaged over each cell of a 35 × 35 nm grid fitted to the survey’s stations.

Number of days of ice were computed using daily sea ice concentrations from Nimbus-7 SMMR and DMSP SSM/I-SSMIS Passive Microwave. Data were retrieved from NSIDC^37^. The total number of days of ice within each year were calculated by presence/absence of sea ice (presence defined by ice concentration > 15%). Remote sensing data with high spatial and temporal resolution were used to obtain open water area for each of the polygons on a yearly basis^38^.

### Data treatment

Environmental conditions were presented by 4 abiotic groups: mean temperature over 10 to 100 m, bottom temperature in °C, number of days with ice and open water area in km^2^. The living ecosystem components were aggregated into 19 biotic groups: zooplankton (mesozooplankton biomass in g dry weight/m^2^, and krill, amphipods and jellyfish biomass in g dry weight/m^2^ estimating a water content of 90% for jellyfish^39^, and 20% for krill and amphipods^40^, megabenthos (commercial benthos, sponges, echinoderms), 0-group (boreal commercial and capelin, herring, polar cod), adult pelagic (herring, capelin, polar cod, blue whiting), and demersal fish (commercial species, other large species, small arctic species, and small boreal species) (Table 1 and supplementary Fig. S2-2–14).

The Barents Sea was divided into 13 polygons based on topography and oceanography (ICES 2018, supplementary Fig. S2-0), and each station was then assigned to a polygon. To ensure equivalent representation of each polygon despite variable coverage in space and time, we bootstrapped 100 times 10 stations per year, polygon and ecosystem component, and calculated the mean value. We thus created 299 time series that were applied in the analyses presented in the paper.

### Analyses

Considering the low number of data points in all time series (18 years) we avoided using correlative analyses to study covariations among all time series. Instead, we focused on identifying periods of similar ecological configurations. All analyses were performed in R^41^. For all analyses except the plot in Fig. 3, each of the 299 time-series were scaled (x − mean(x)/sd(x)) to avoid spatial gradients masking temporal ones. We calculated the total absolute variability by summing the absolute difference of each year’s value to the mean of the time series. To identify temporal phases in environmental conditions, we used hierarchical clustering with temporal contiguity constraints on mean 10–100 m temperature across the whole Barents Sea, using the “constr.hclust” function from the adespatial R package^20^. The best model was determined using Euclidean distance, and the “complete” clustering method.

To explore the space–time dynamics of the 23 groups, for each of them we calculated the mean raw values (i.e. not scaled) over each of the 3 periods, for each polygon. We then ran a clustering analysis for 6 assemblages of 3 to 4 ecosystem components. To do so, first we ran a PCA where individuals are mean values for each polygon-period couple. Then, we used the function HCPC from FactomineR R package^42^ which runs a hierarchical analysis consolidated by kmeans clustering. This analysis groups together polygons that have similarly high/low values of ecosystem components among the three periods (Supplementary material, S3).

## Supplementary Information

Supplementary Information 1.

Supplementary Information 2.

Supplementary Information 3.

## Data Availability

The datasets generated and/or analysed during the current study are in the process of being made available in the Norwegian Marine Data Centre (NMD) repository. The datasets are at present available on request to the corresponding author (elena.eriksen@hi.no).
