# Supplementary material for: The living Barents Sea response to peak-warming and subsequent cooling
Source: Sci Rep. 2025 Apr 15;15:13008. doi: 10.1038/s41598-025-96964-x (PMC12000424; doi:10.1038/s41598-025-96964-x)
Supplement: Supplementary file 1 — Supplementary Information 1. [file 41598_2025_96964_MOESM1_ESM.docx]

The document contains main results with figures for all ecosystem components and environmental parameters studied**
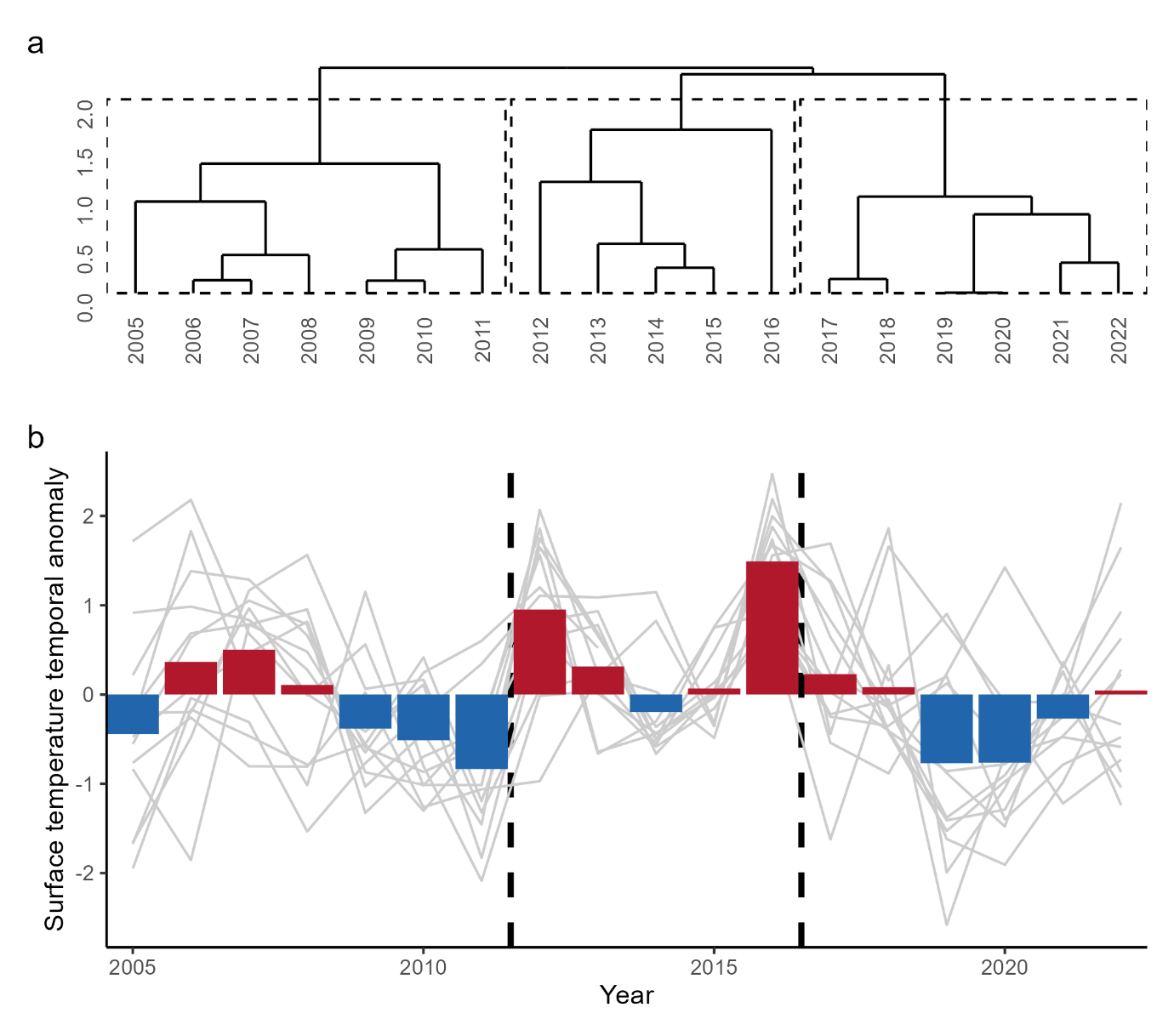
**

Fig. S1-1. a. Three time periods characterized by different thermal conditions identified through temporally constrained hierarchical clustering of the average bottom temperature. b. Time series of temporal anomalies for 2005-2022 (below). The bars show average annual temperatures for all polygons while the grey lines show annual temperature for each polygon. The three identified time periods are separated by dashed vertical lines and averaged temperature for each period with associated standard deviation is given on top.


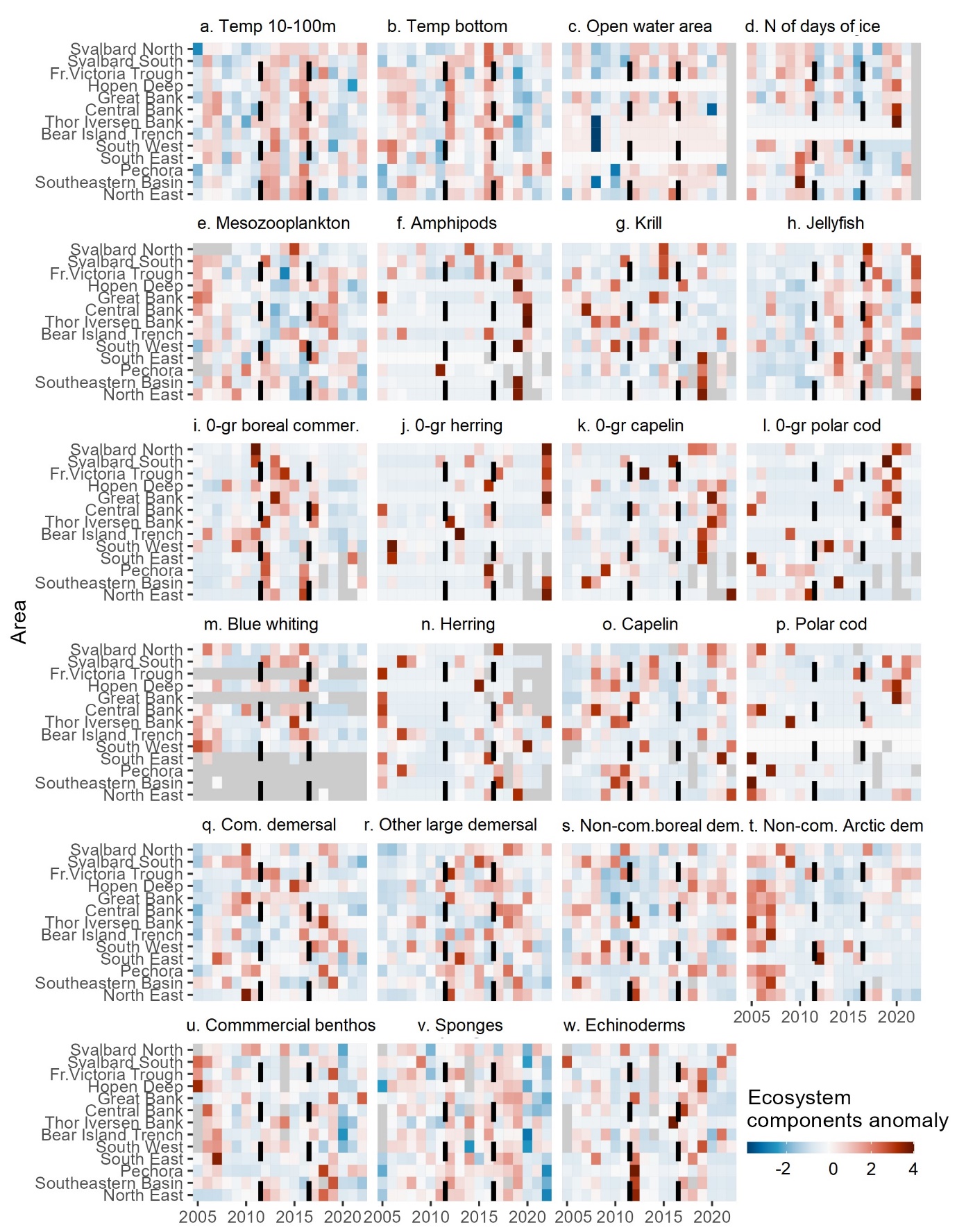


Fig. S1-2a-w. Anomaly heatmaps of the BS ecosystem. Polygons are ordered from northwestern areas (top) to northeastern areas (bottom). Anomalies are calculated relative to the average in each polygon over 2005-2022. Missing data are in grey.

Below a mean biomass of studied biotic groups shown by sub-area and period.

**
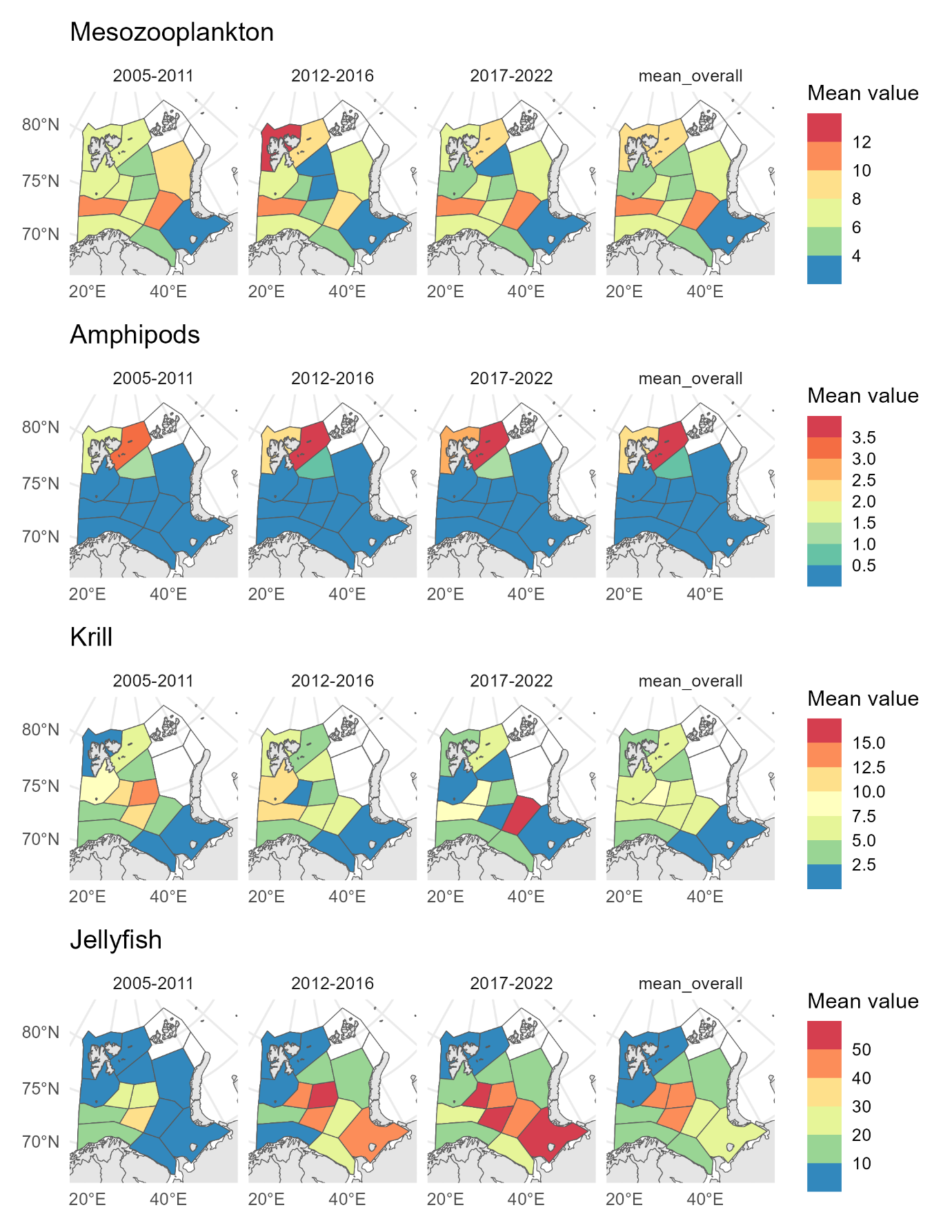
** Fig. S1-3a-d


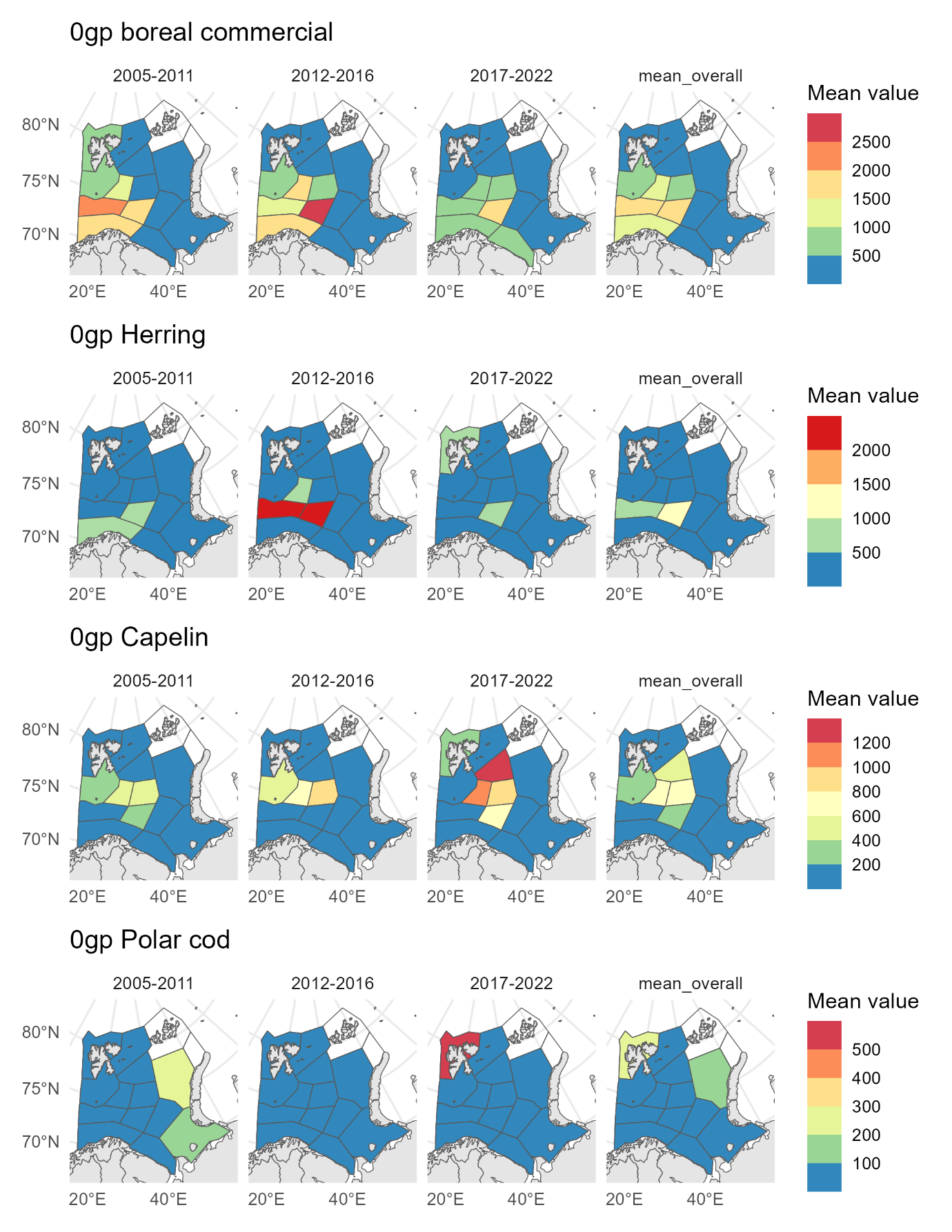


Fig. S1-4a-d


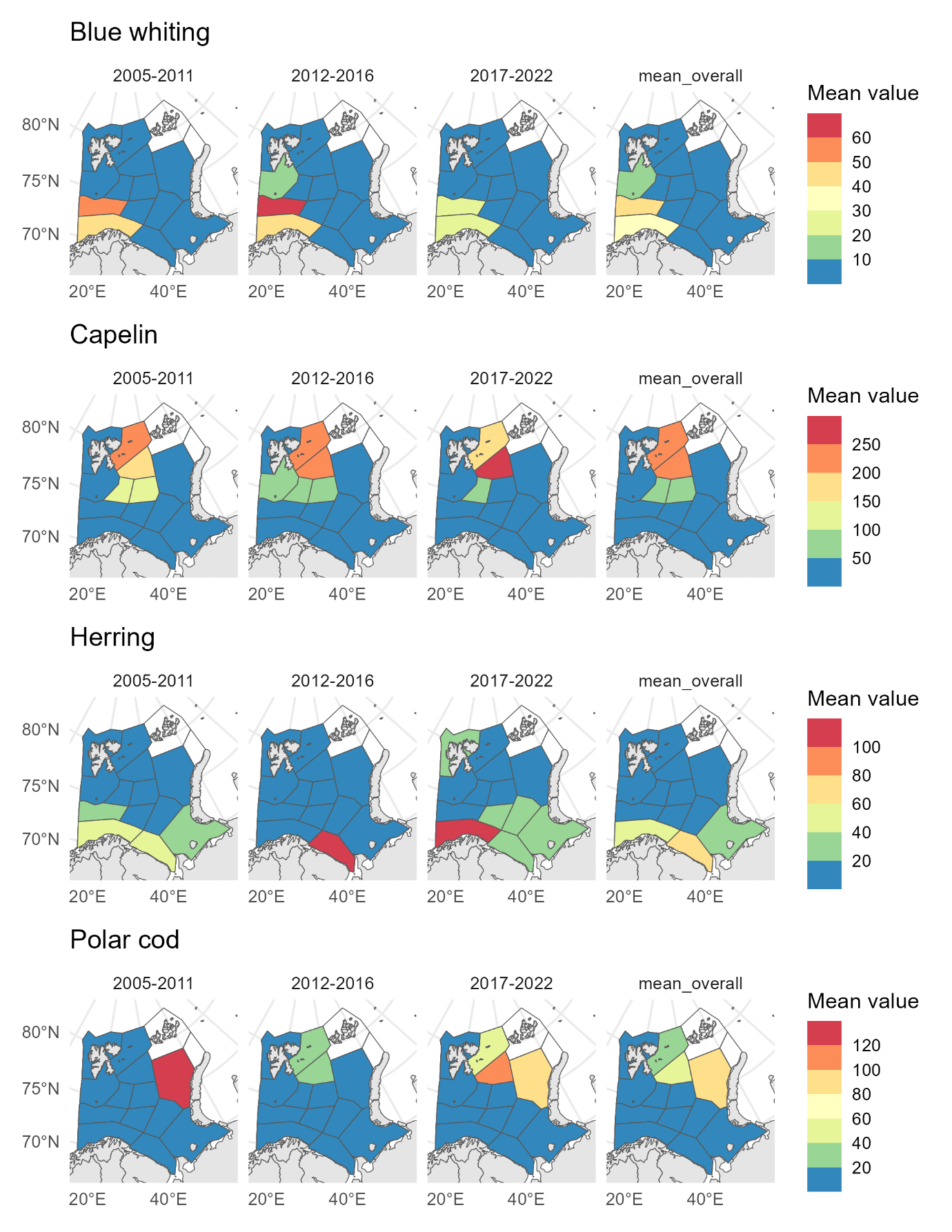


Fig. S1-5a-d


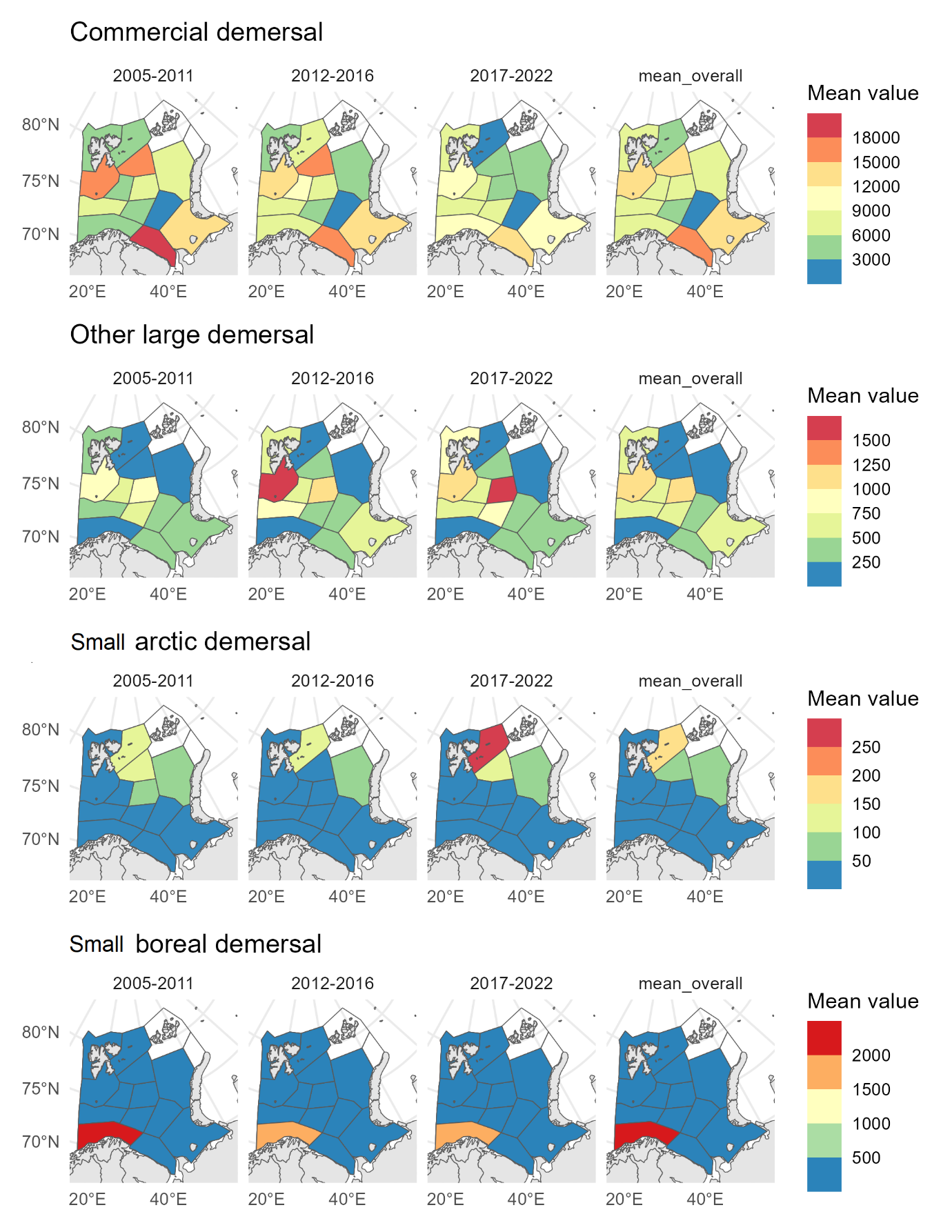


Fig. S1-6a-d


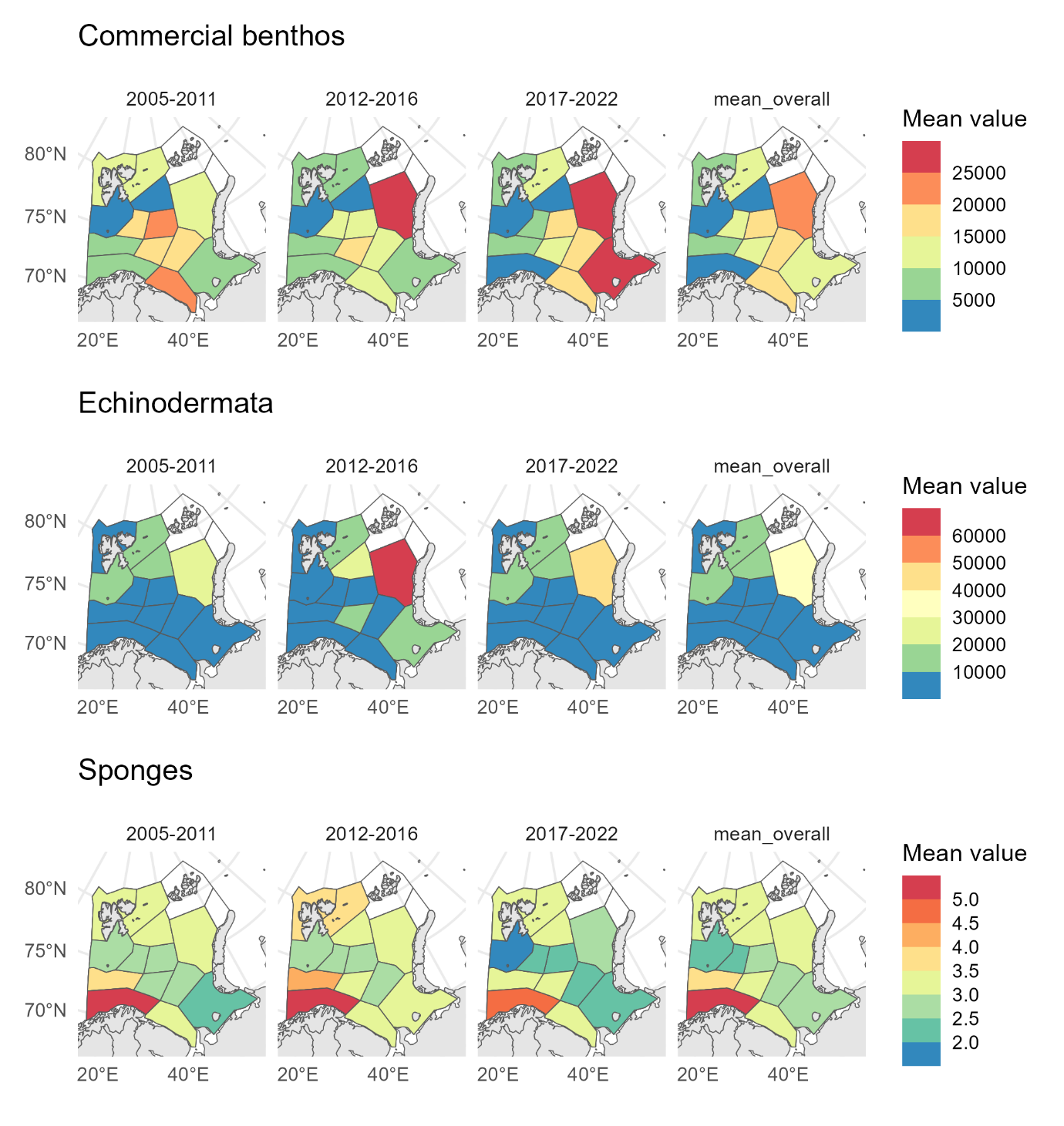


Fig. S1-7a-c


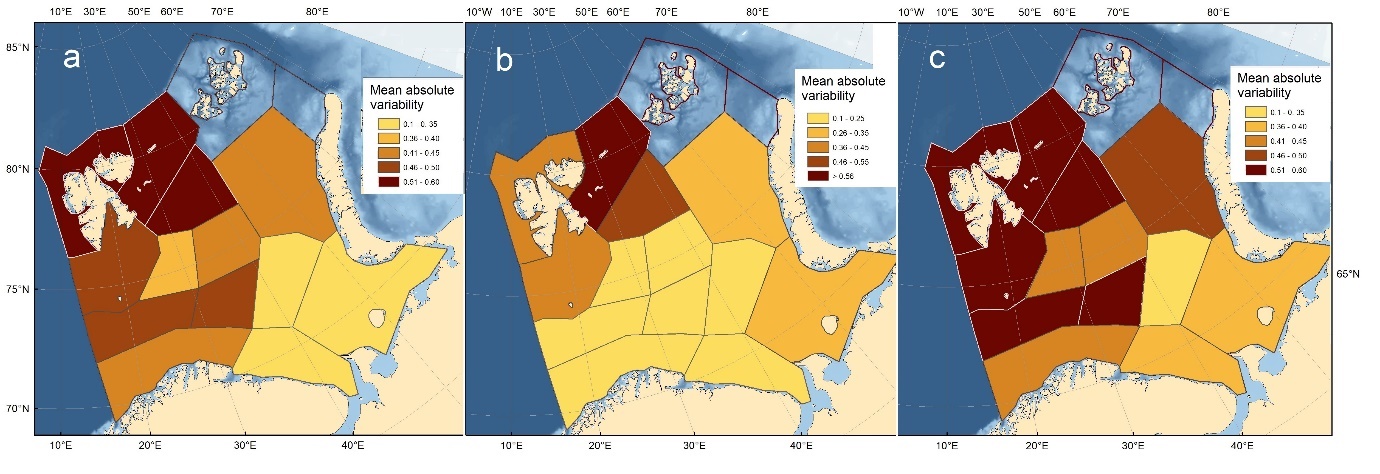


Fig. S1-8a-c. Most variable polygons: (a) overall magnitude of change across polygons (sum of the yearly standardized absolute deviation from the mean) (b) in environment (c) and biology.


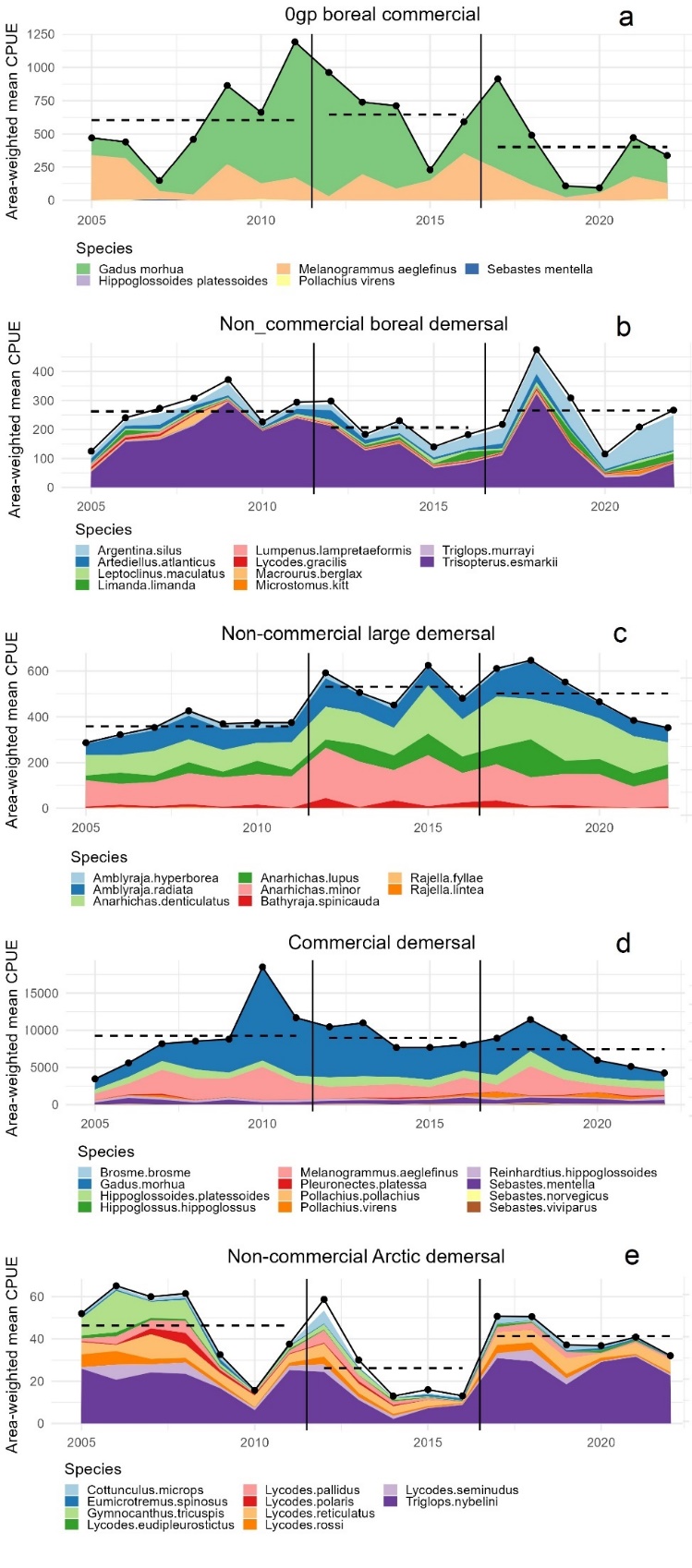


Fig. S1-9a-e. Area-weighted mean catches standardized by effort (CPUE) for different assemblages. Advected 2nd TL plankton - weight of jellyfish, krill, and amphipods were reduced by water content (0,04% for jellyfish and 0,2 for krill and amphipods) to make them comparable. Advected low TL-plankton consists of mesozooplankton. Advected pelagic showed by repunish colors and include all 0-group fishes; swimming pelagic (reddish) include pelagic species such as capelin, polar cod, young herring, and blue whiting. Demersal community (greyish) include both commercial, large demersal and non-commercial small fishes. Benthos community (orangish) includes both commercial benthos, echinoderms and sponges.
