# Supplementary material for: The living Barents Sea response to peak-warming and subsequent cooling
Source: Sci Rep. 2025 Apr 15;15:13008. doi: 10.1038/s41598-025-96964-x (PMC12000424; doi:10.1038/s41598-025-96964-x)
Supplement: Supplementary file 2 — Supplementary Information 2. [file 41598_2025_96964_MOESM2_ESM.docx]

**Overview of time series for 24 ecosystem components for each of 13 polygons for the period 2005-2022.**


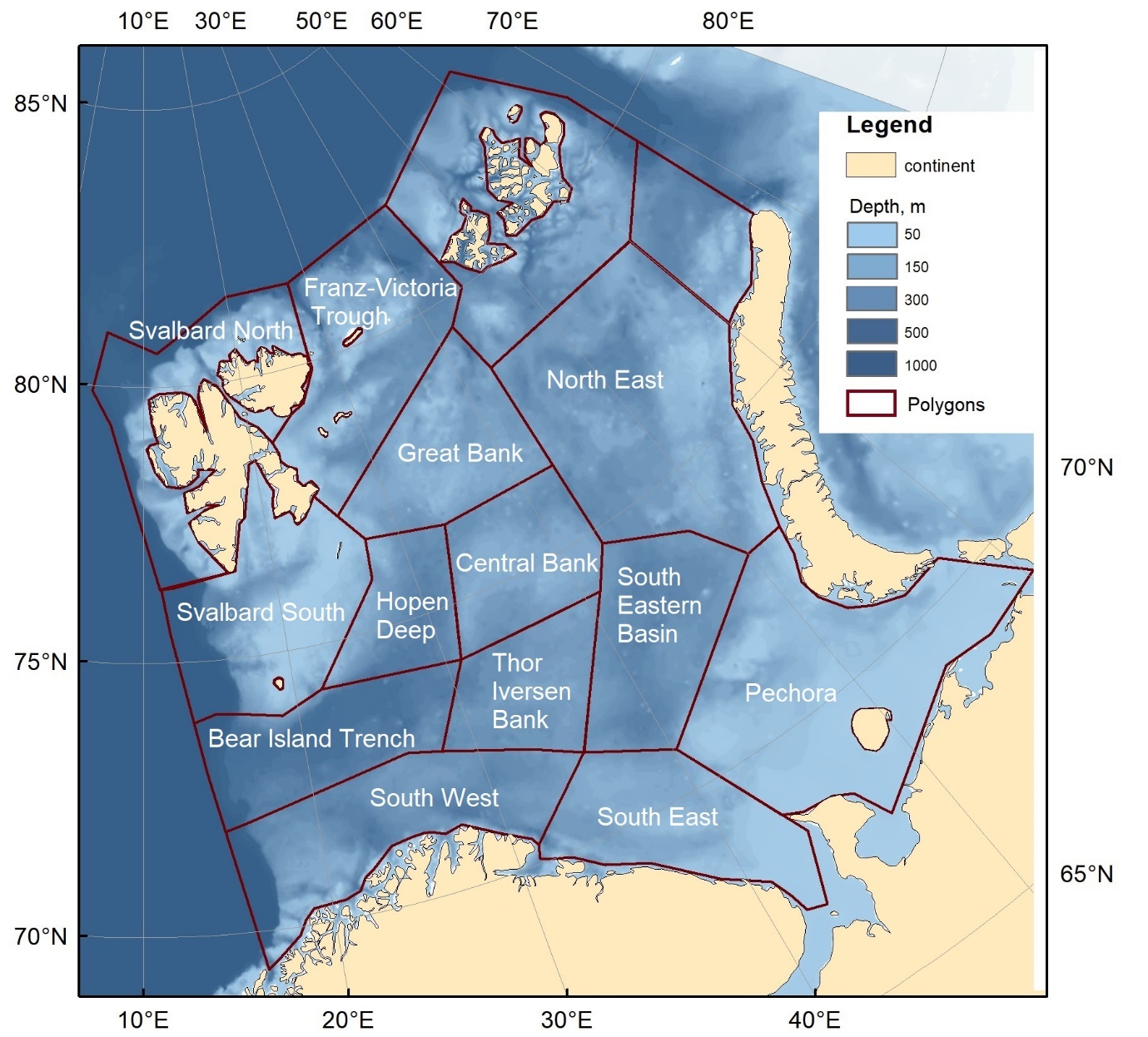


Fig. S2-0. The Barents Sea with polygons


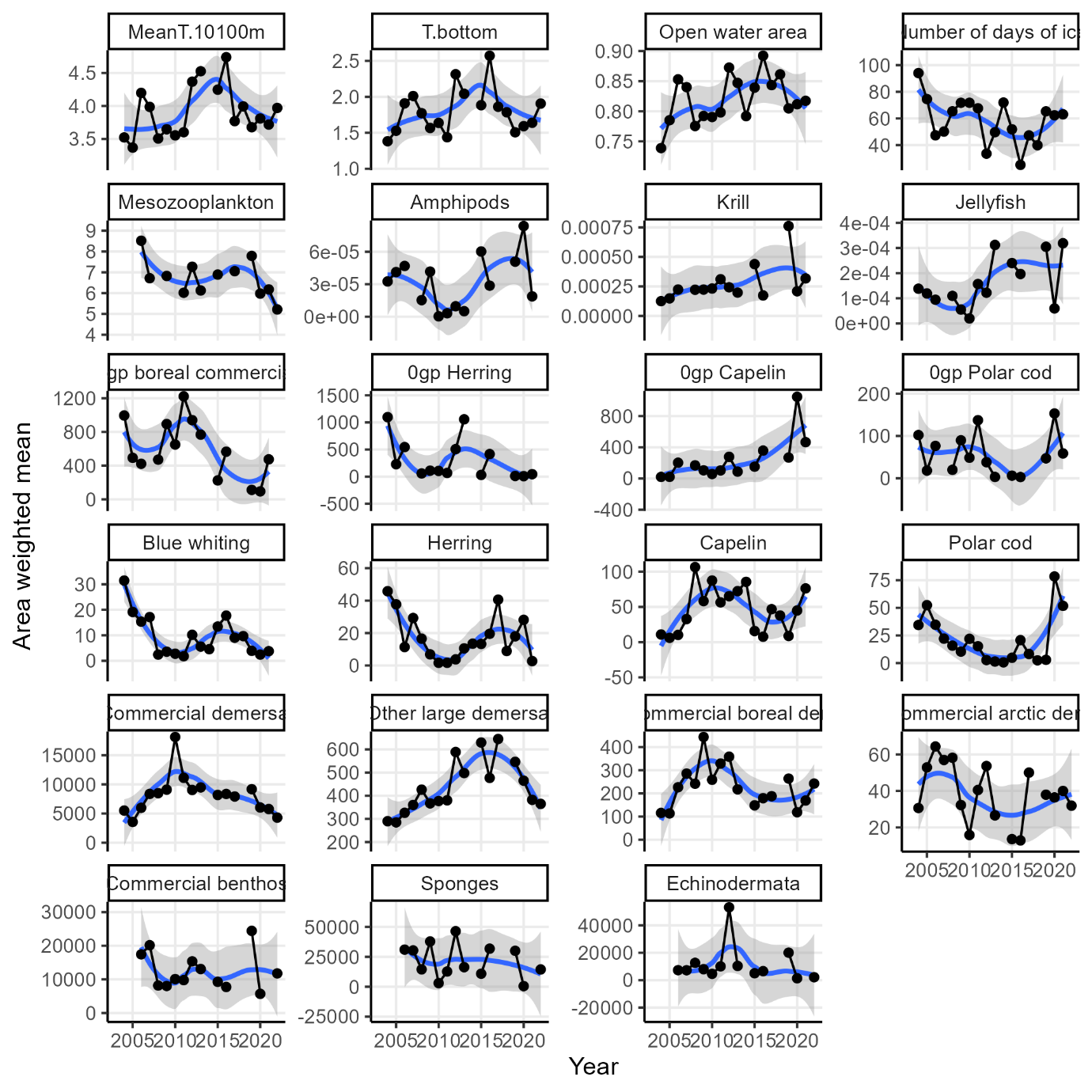


Fig.S2-1. Area-weighted mean catches standardized by effort (CPUE) for different ecosystem components for the period 2005-2022. The observed values for different assemblages are displayed, including those for mesozooplankton (g dry weight/m^2^ ), while krill, amphipods, jellyfish and fish (kg/km^2^) and benthos (g/km^2^)

Fig. S2-2-14 show mean value for all ecosystem components. Polygons are sorted from northwestern areas (top) to northeastern areas (bottom).


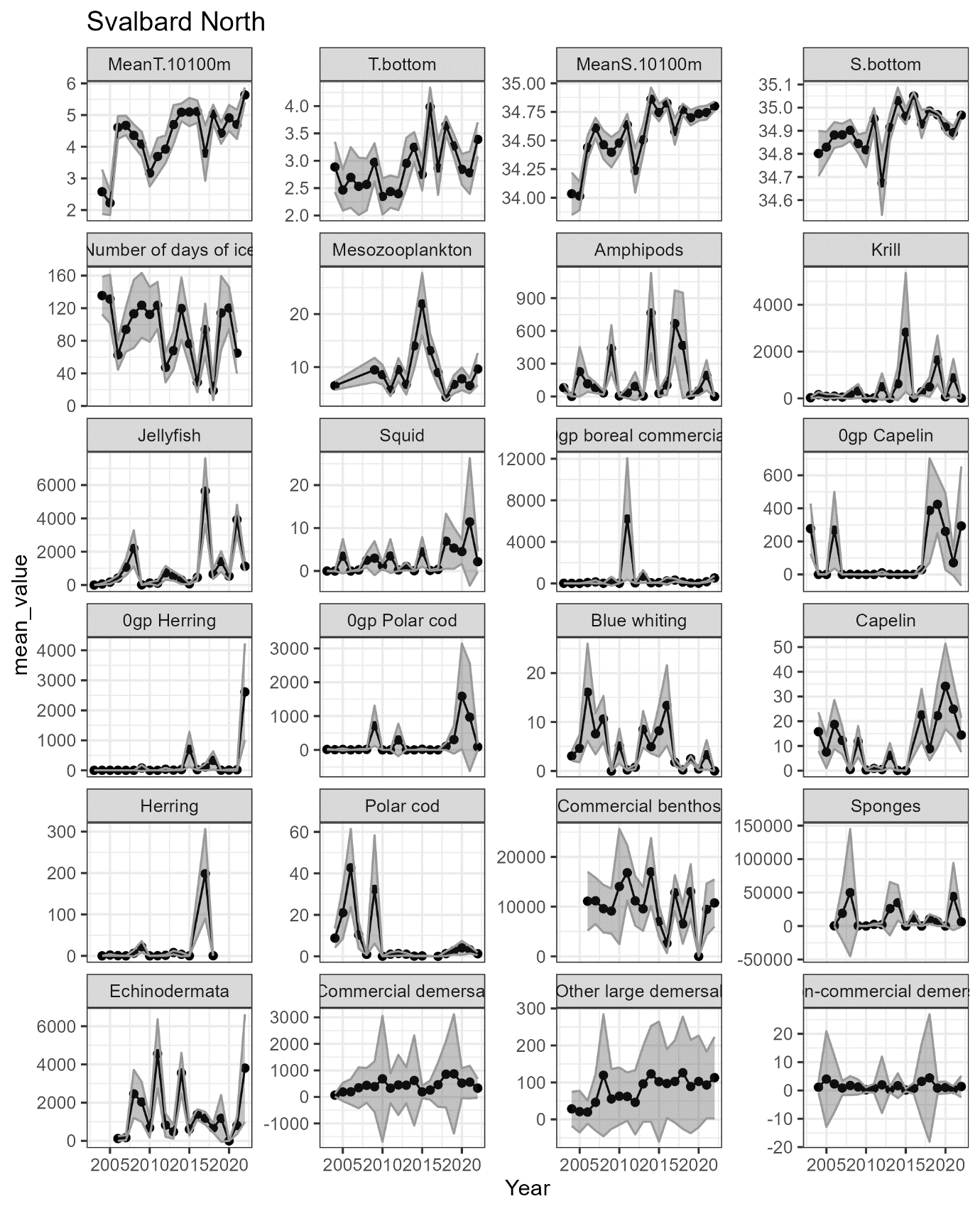


Fig S2-2. Time series for the Svalbard North polygon. The observed values for different assemblages are displayed, including those for mesozooplankton (g dry weight/m^2^ ), while krill, amphipods, jellyfish and fish (kg/km^2^) and benthos (g/km^2^)


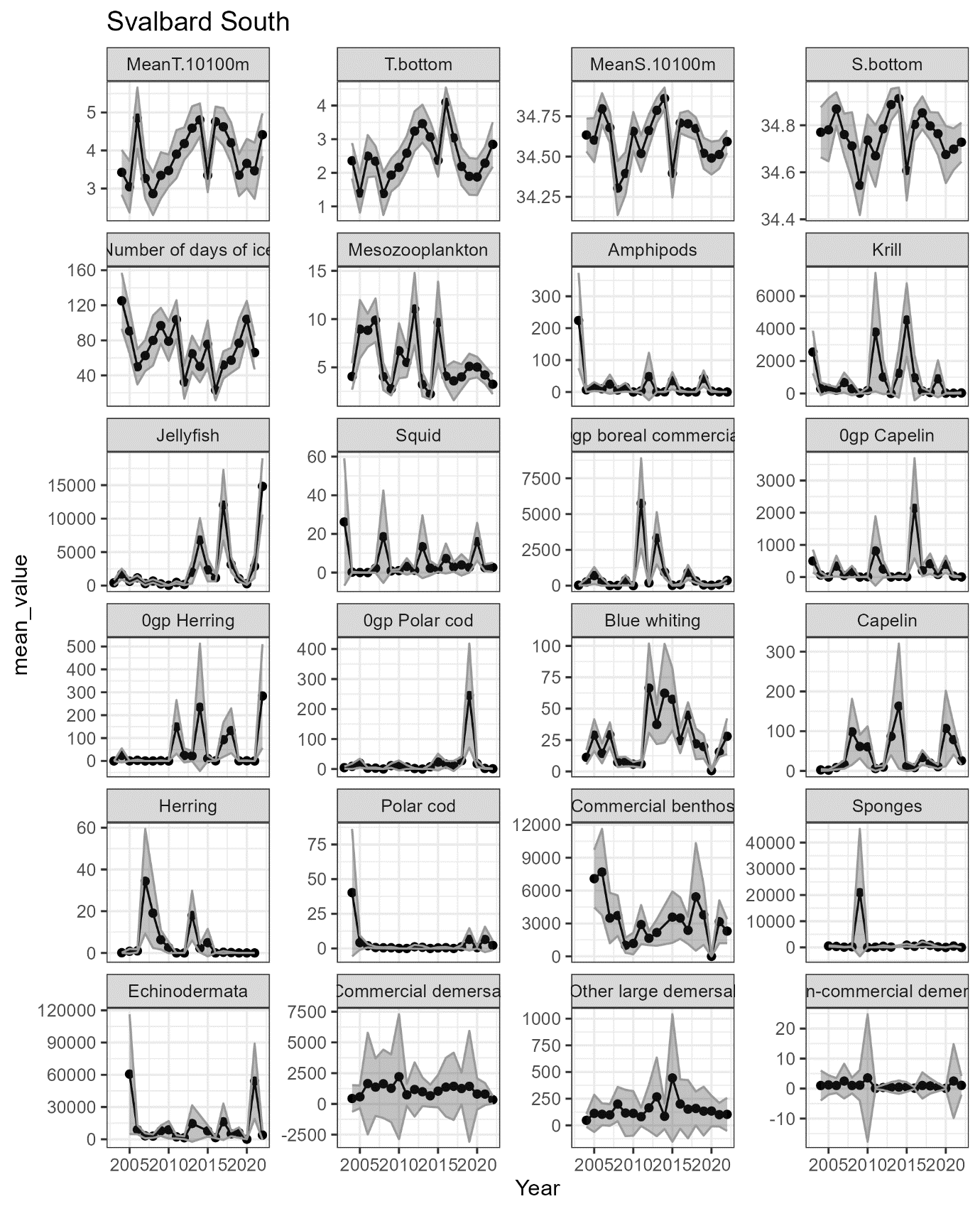
 Fig S2-3. Time series for the Svalbard South polygon. The observed values for different assemblages are displayed, including those for mesozooplankton (g dry weight/m^2^ ), while krill, amphipods, jellyfish and fish (kg/km^2^) and benthos (g/km^2^).


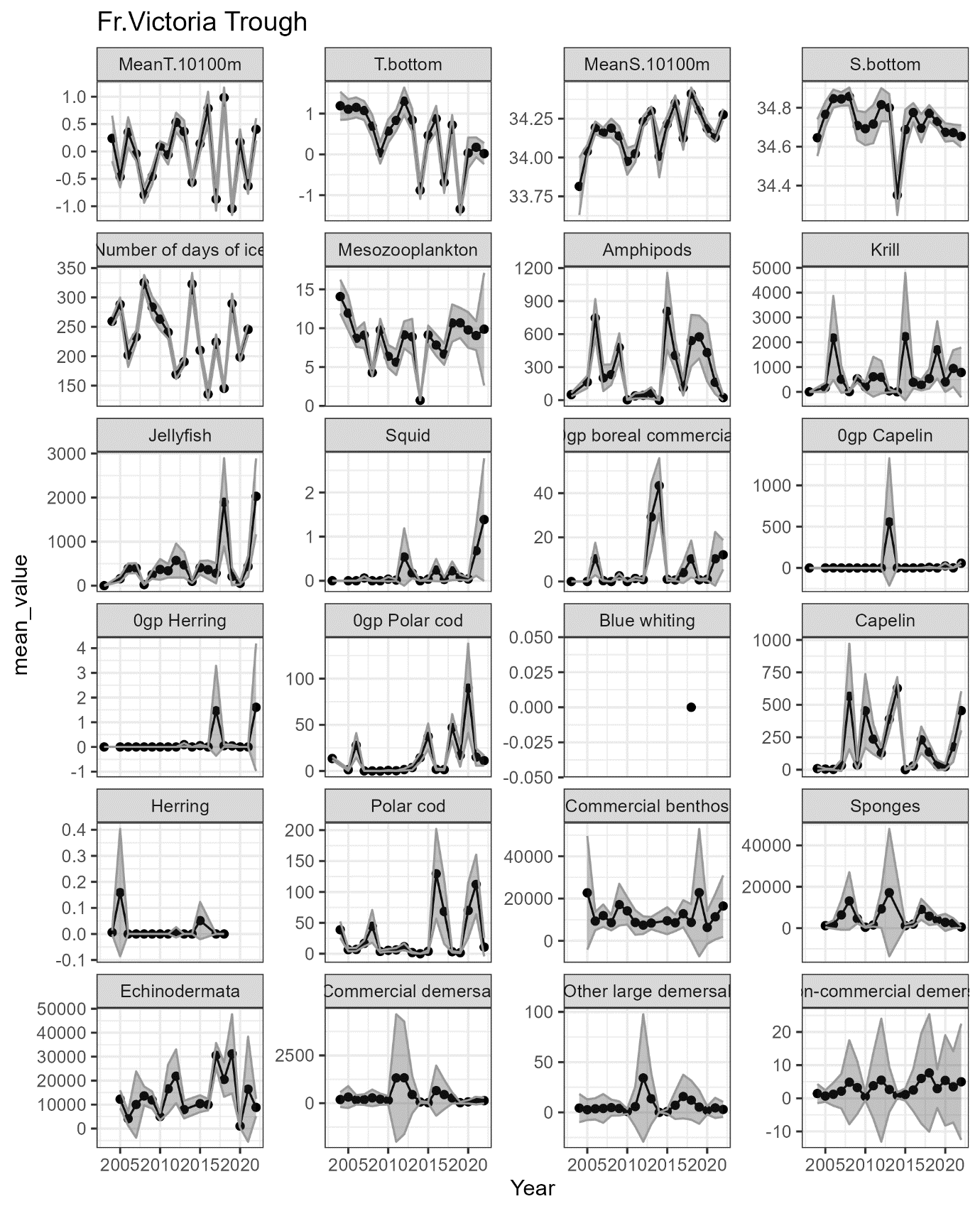
 Fig S2-4. Time series for the Fr. Victoria Trough polygon. The observed values for different assemblages are displayed, including those for mesozooplankton (g dry weight/m^2^ ), while krill, amphipods, jellyfish and fish (kg/km^2^) and benthos (g/km^2^)


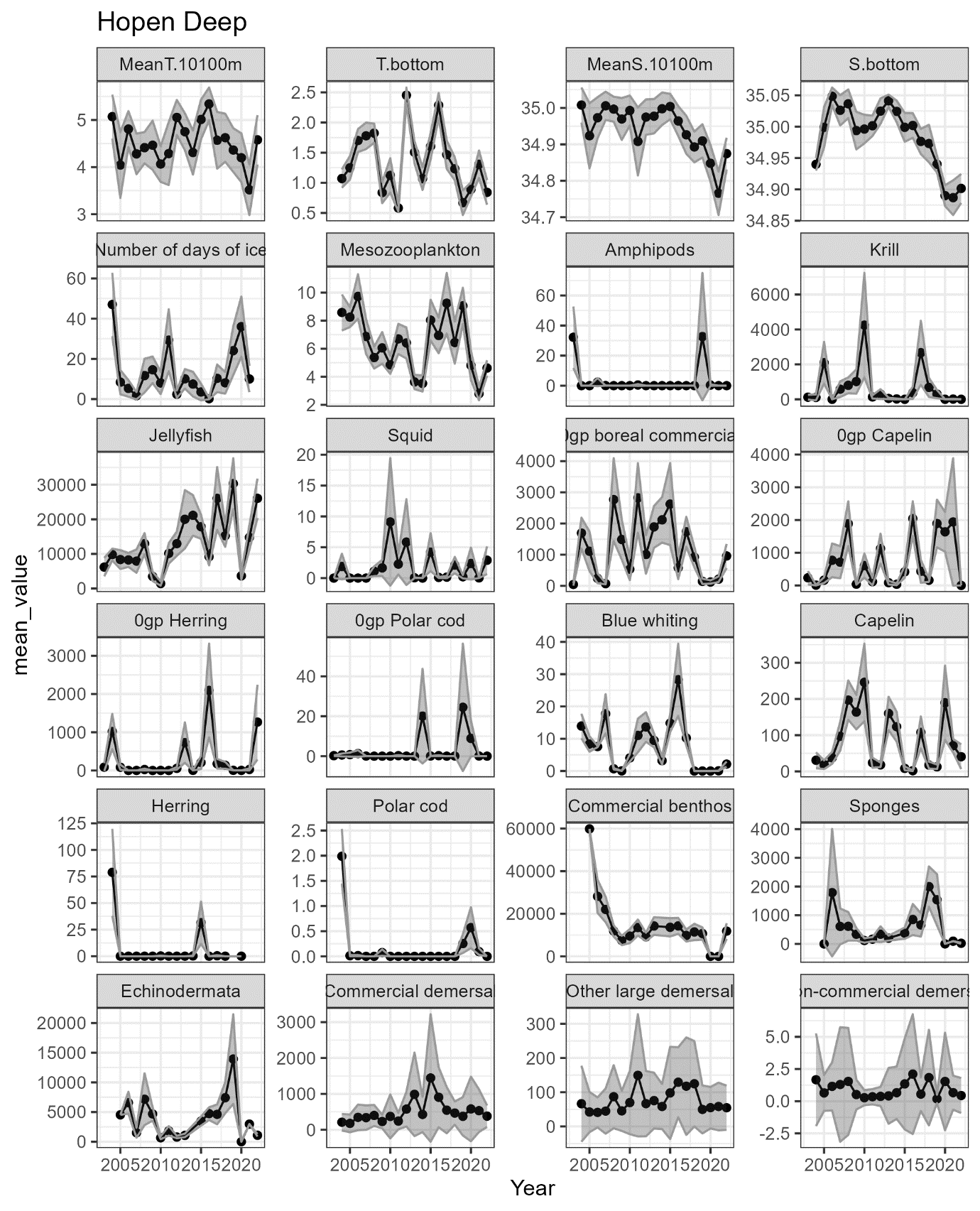
 Fig S2-5. Time series for the Hopen Deep polygon. The observed values for different assemblages are displayed, including those for mesozooplankton (g dry weight/m^2^ ), while krill, amphipods, jellyfish and fish (kg/km^2^) and benthos (g/km^2^)


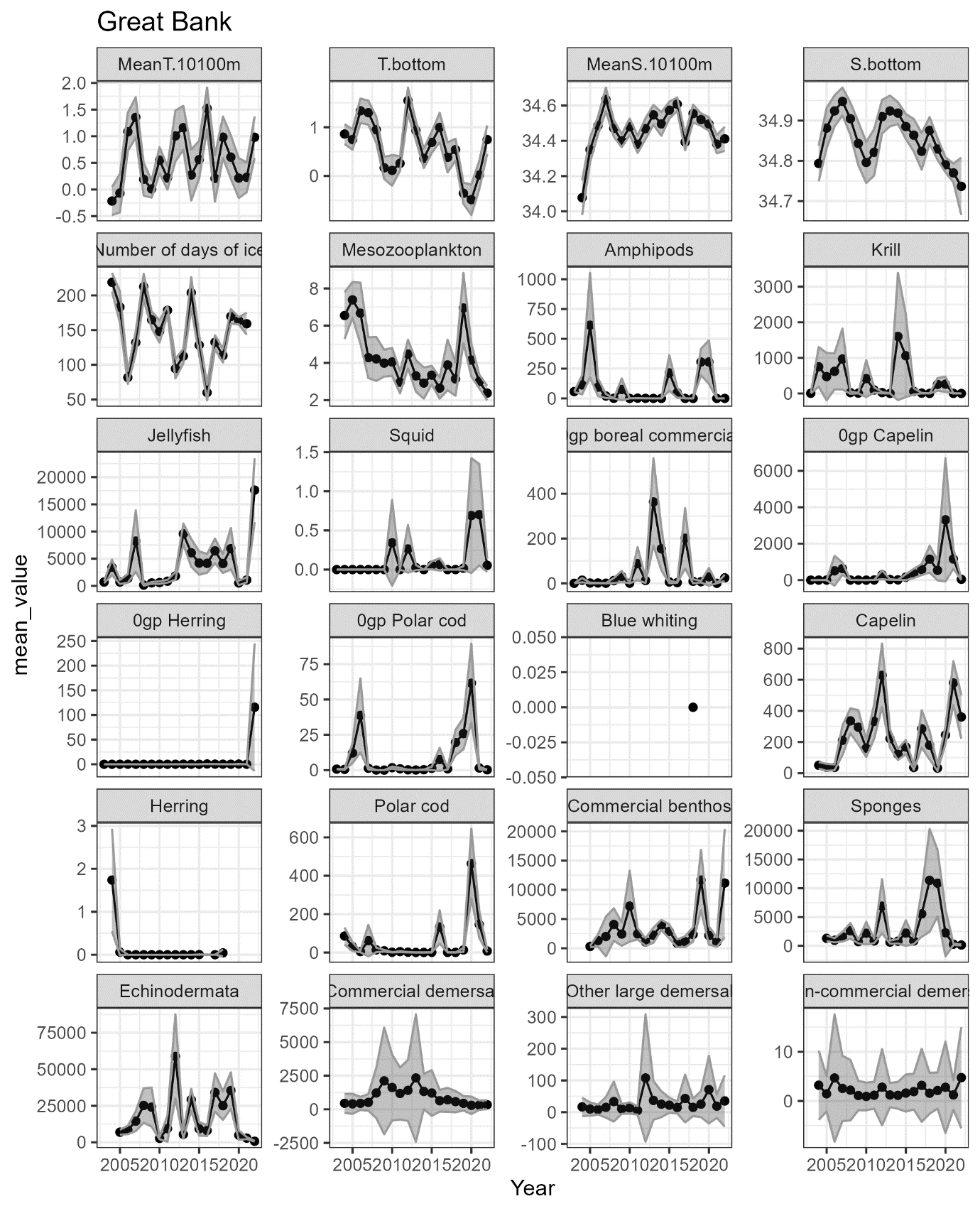


Fig S2-6. Time series for the Great Bank polygon. The observed values for different assemblages are displayed, including those for mesozooplankton (g dry weight/m^2^ ), while krill, amphipods, jellyfish and fish (kg/km^2^) and benthos (g/km^2^)


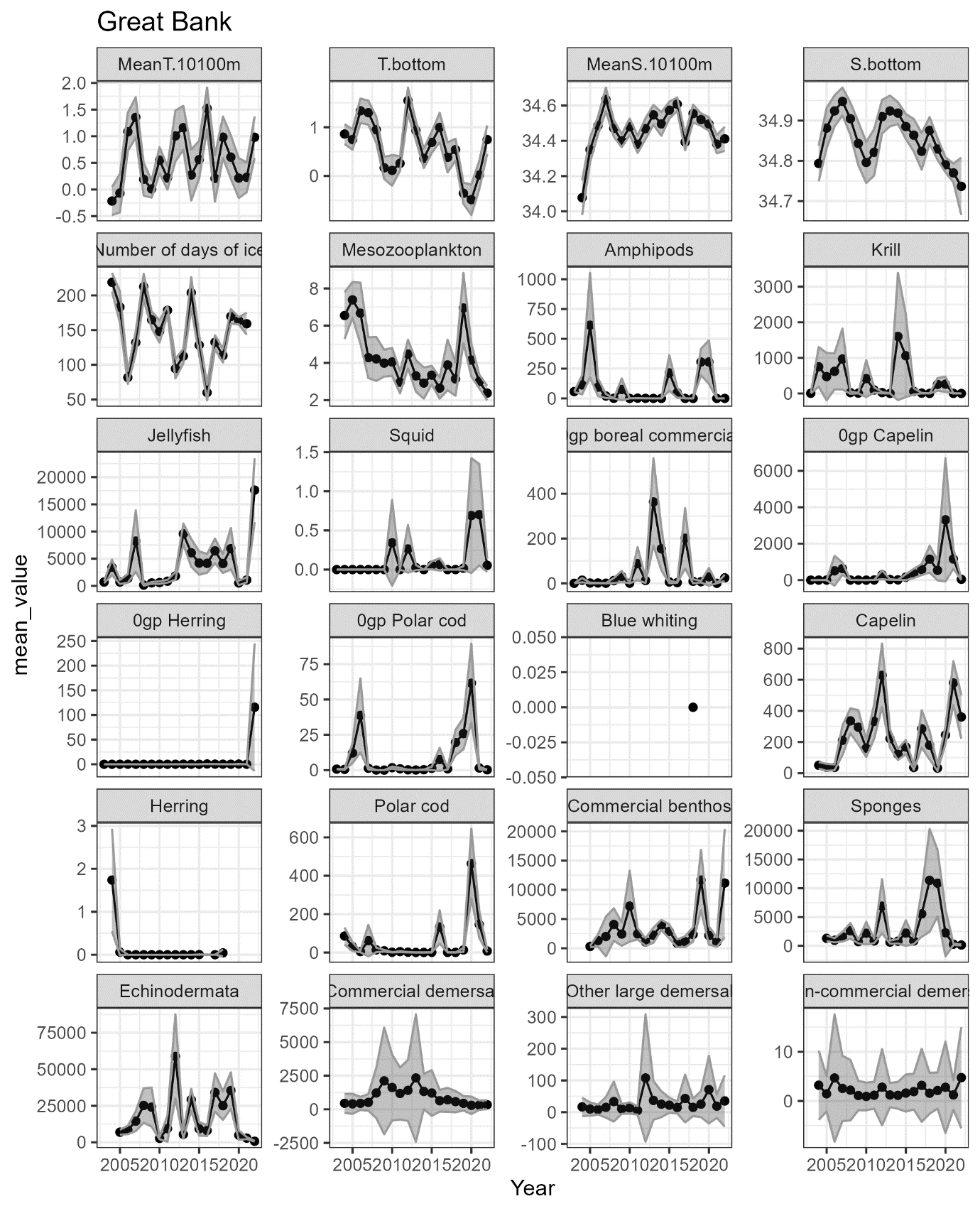
 Fig S2-7. Time series for the Central Bank polygon. The observed values for different assemblages are displayed, including those for mesozooplankton (g dry weight/m^2^ ), while krill, amphipods, jellyfish and fish (kg/km^2^) and benthos (g/km^2^)


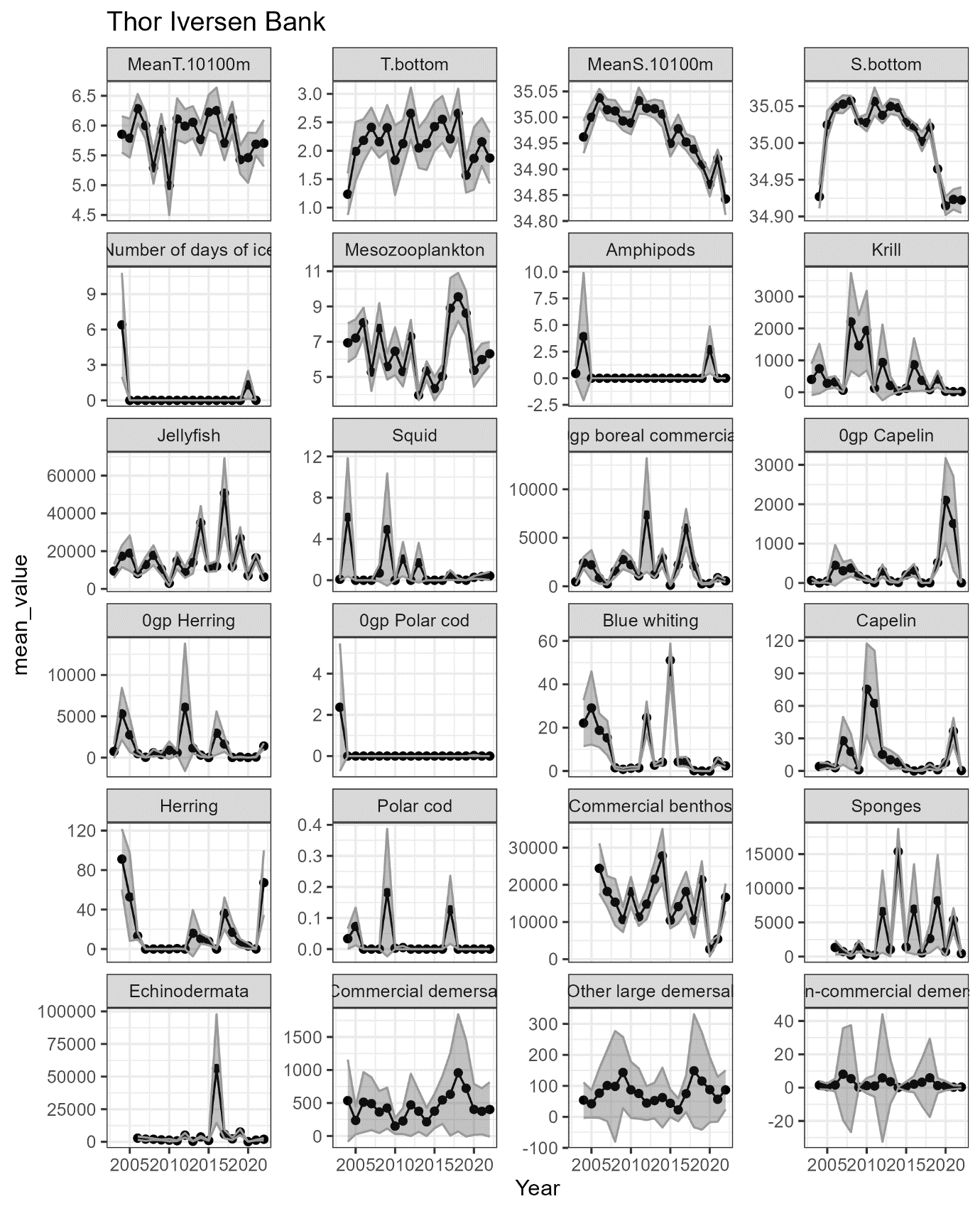
 Fig S2-8. Time series for the Thor Iversen Bank polygon. The observed values for different assemblages are displayed, including those for mesozooplankton (g dry weight/m^2^ ), while krill, amphipods, jellyfish and fish (kg/km^2^) and benthos (g/km^2^)


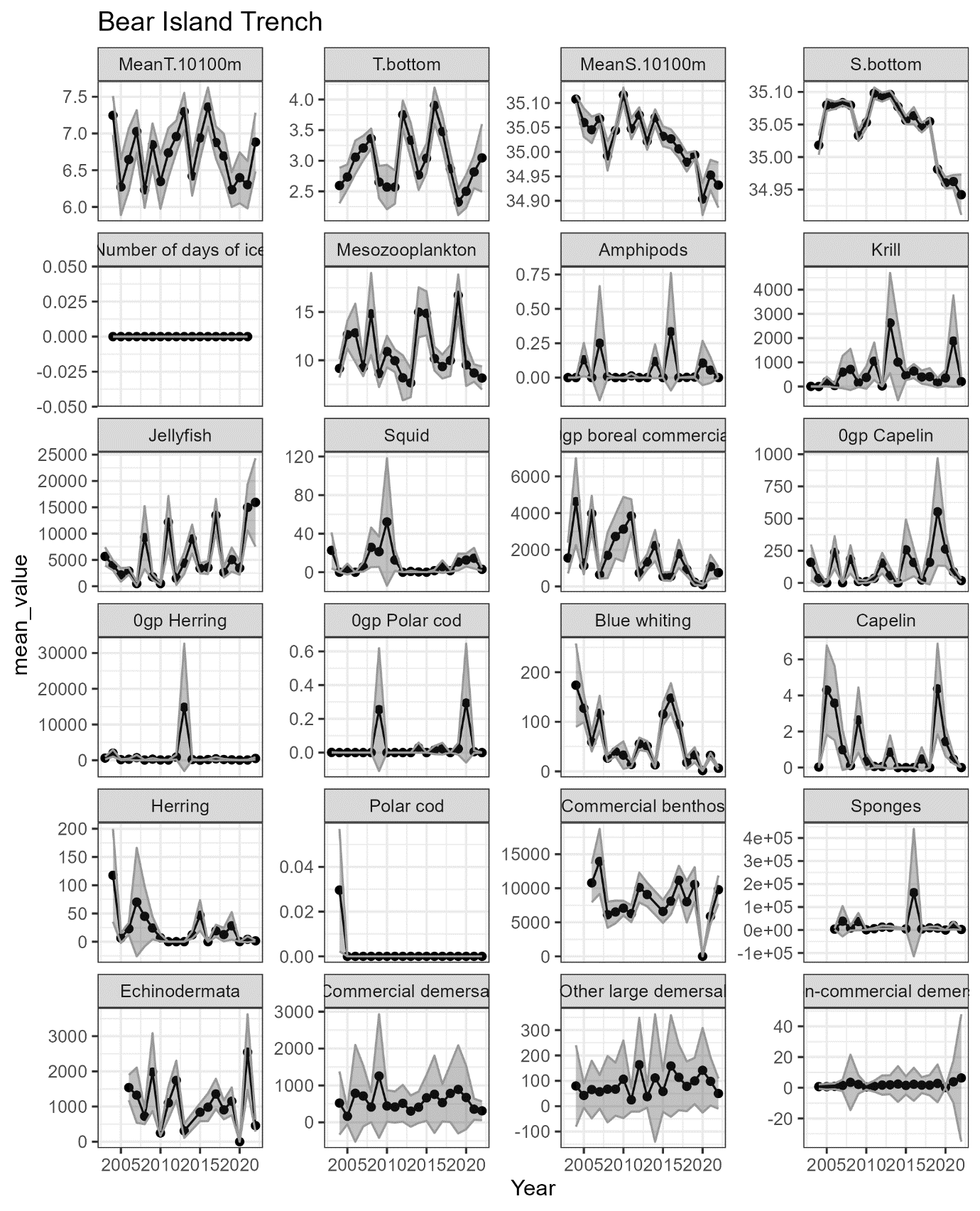
 Fig S2-9. Time series for the Bear Island Trench polygon. The observed values for different assemblages are displayed, including those for mesozooplankton (g dry weight/m^2^ ), while krill, amphipods, jellyfish and fish (kg/km^2^) and benthos (g/km^2^)


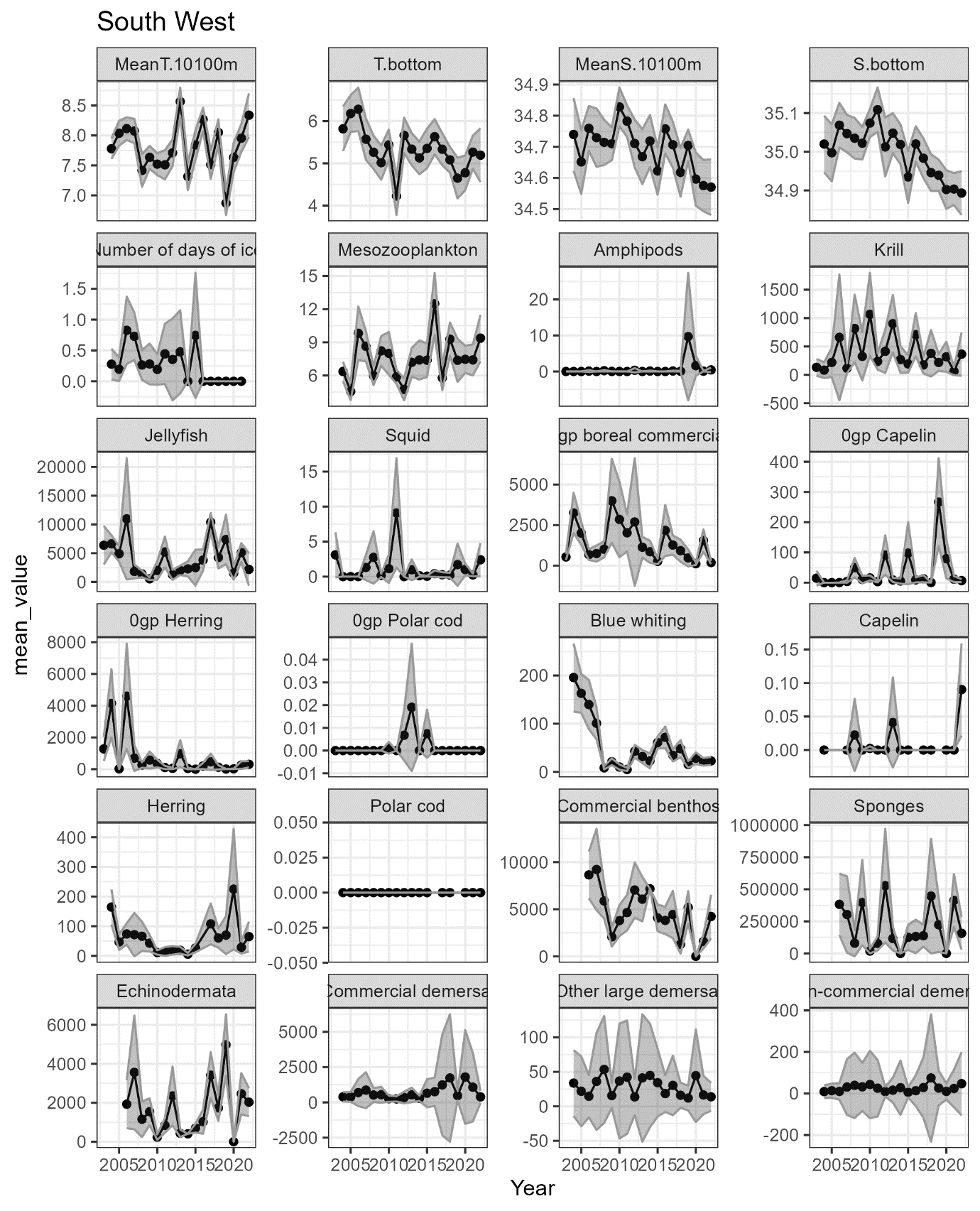
 Fig S2-10. Time series for the South West polygon. The observed values for different assemblages are displayed, including those for mesozooplankton (g dry weight/m^2^ ), while krill, amphipods, jellyfish and fish (kg/km^2^) and benthos (g/km^2^)


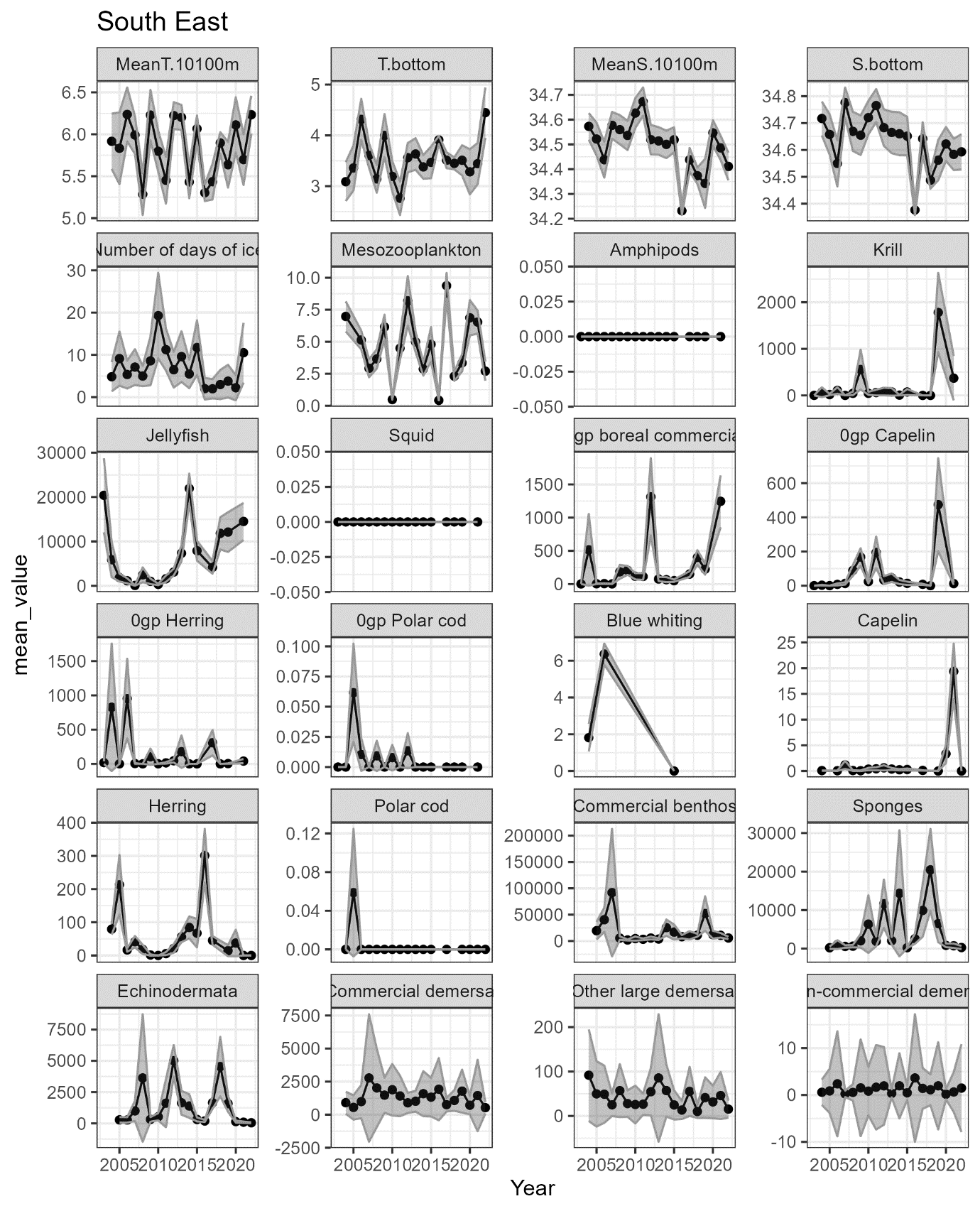


Fig S2-11. Time series for the South East polygon. The observed values for different assemblages are displayed, including those for mesozooplankton (g dry weight/m^2^ ), while krill, amphipods, jellyfish and fish (kg/km^2^) and benthos (g/km^2^)


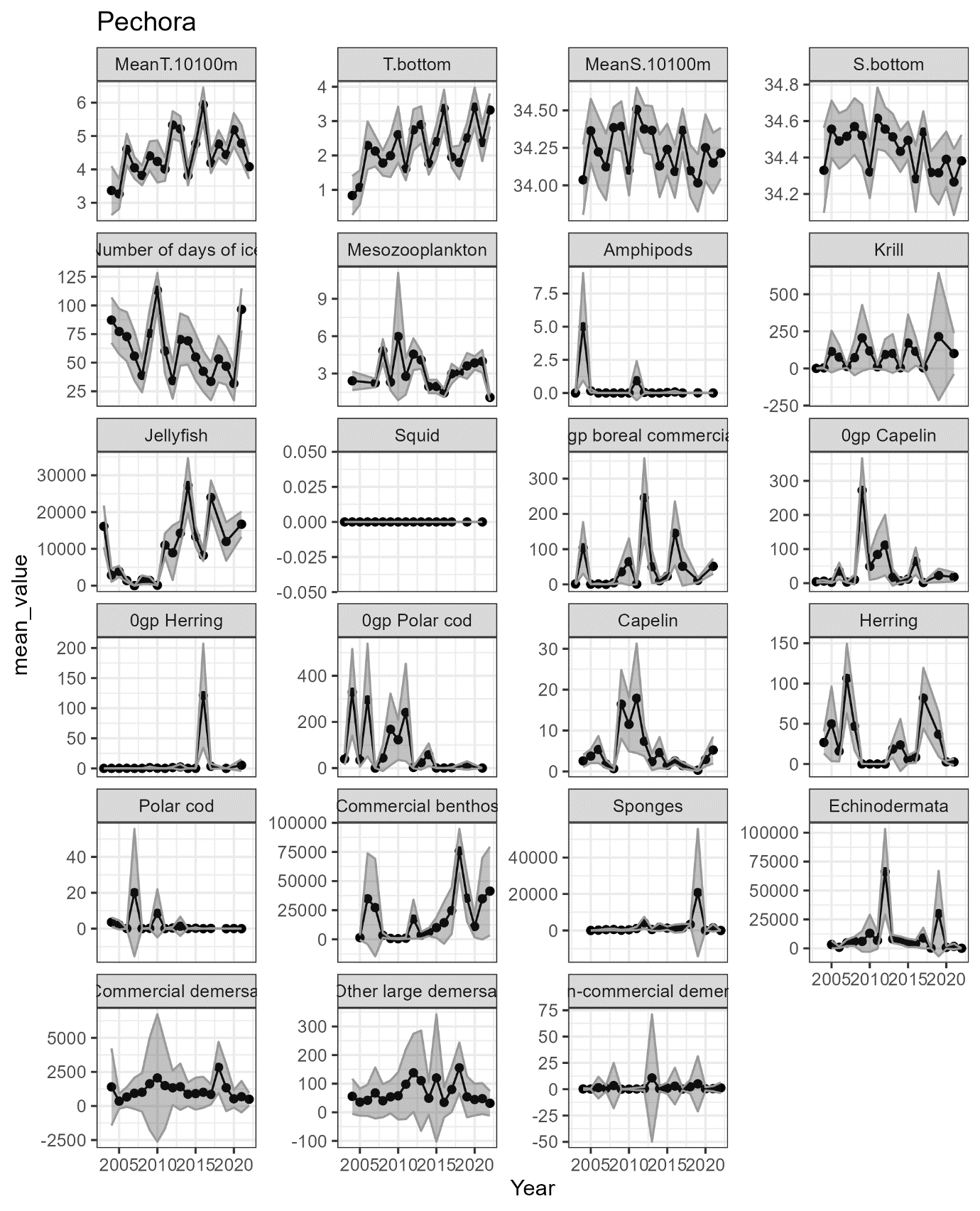


Fig S2-12. Time series for the Pechora polygon. The observed values for different assemblages are displayed, including those for mesozooplankton (g dry weight/m^2^ ), while krill, amphipods, jellyfish and fish (kg/km^2^) and benthos (g/km^2^)


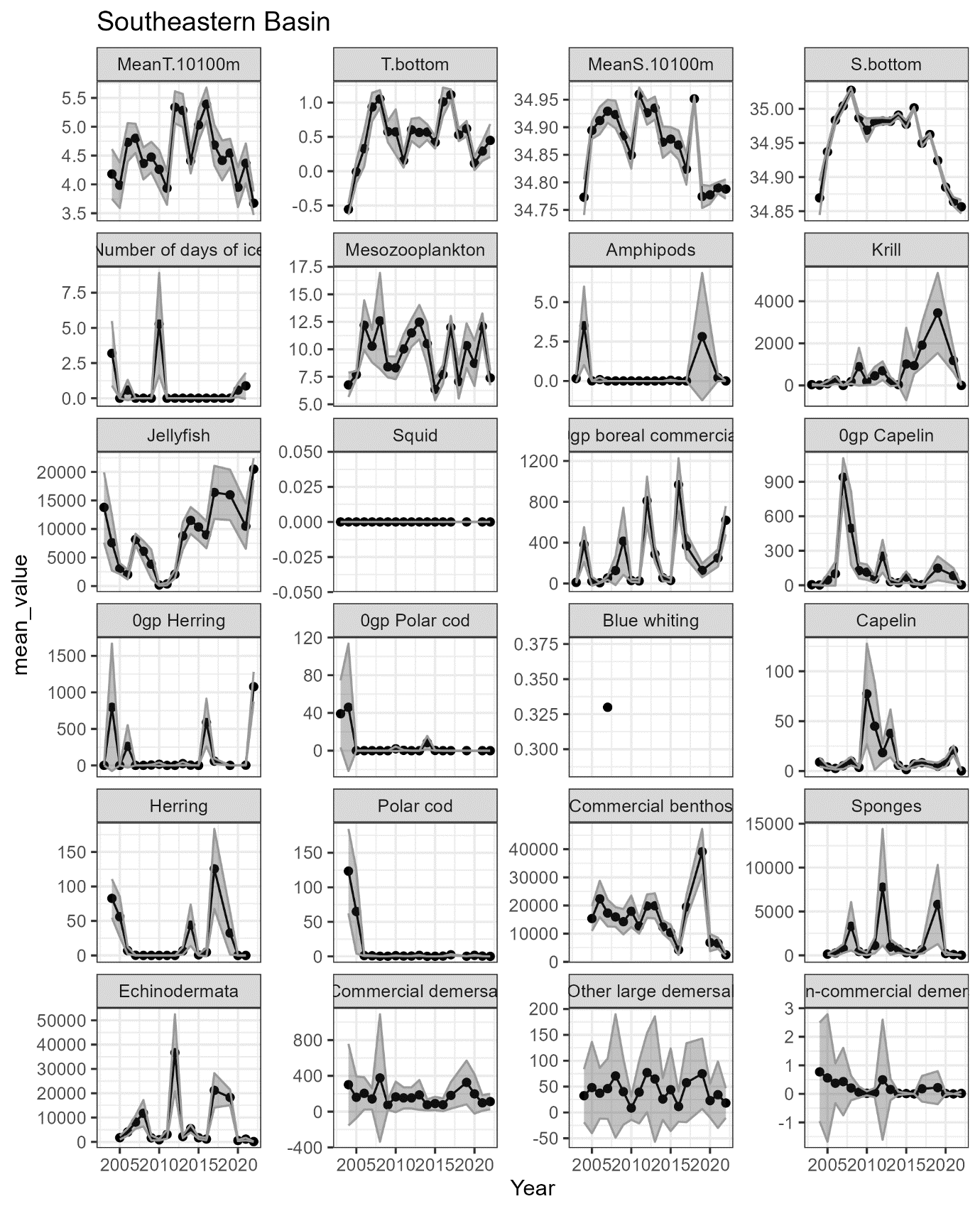
 Fig S2-13. Time series for the Southeastern Basin polygon. The observed values for different assemblages are displayed, including those for mesozooplankton (g dry weight/m^2^ ), while krill, amphipods, jellyfish and fish (kg/km^2^) and benthos (g/km^2^)


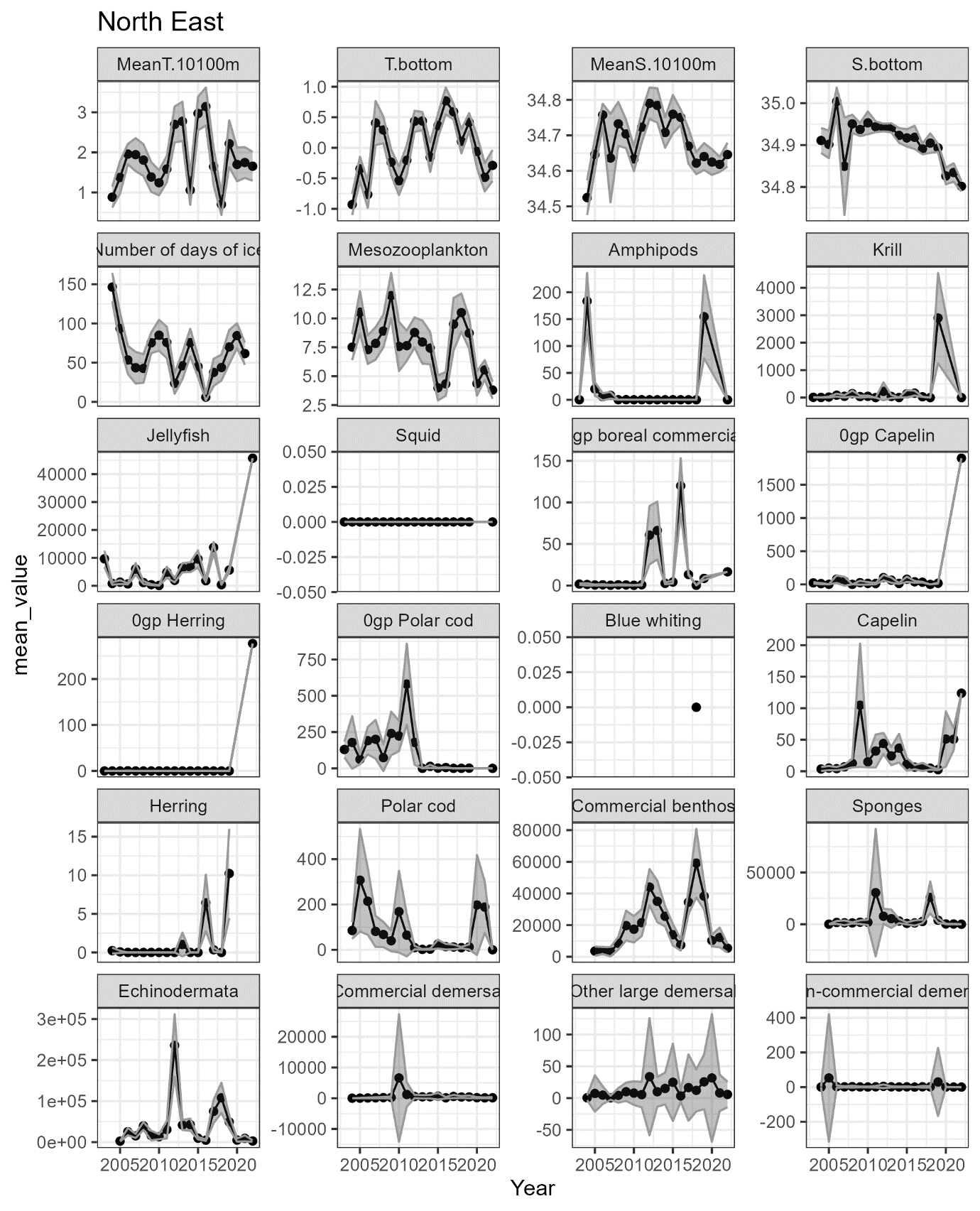
 Fig S2-14. Time series for the North East polygon. The observed values for different assemblages are displayed, including those for mesozooplankton (g dry weight/m^2^ ), while krill, amphipods, jellyfish and fish (kg/km^2^) and benthos (g/km^2^)
