# Supplementary material for: The living Barents Sea response to peak-warming and subsequent cooling
Source: Sci Rep. 2025 Apr 15;15:13008. doi: 10.1038/s41598-025-96964-x (PMC12000424; doi:10.1038/s41598-025-96964-x)
Supplement: Supplementary file 3 — Supplementary Information 3. [file 41598_2025_96964_MOESM3_ESM.docx]

**Integrated analyses for the living Barents Sea.**

To explore the space-time dynamics of the 23 ecosystem components, for each of them, we calculated the mean raw values (i.e. not scaled) over each of the 3 periods, for each polygon. We then ran a clustering analysis for 6 groups of 3 to 4 ecosystem components. To do so, first we ran a PCA where individuals are mean values for each polygon-period couple. Then, we used the function HCPC from FactomineR R package (Le et al., 2008) which runs a hierarchical analysis consolidated by kmeans clustering. This analysis groups together polygons that have similarly high/low values of ecosystem components among the three periods (Fig. SM3-1).

**The initial period (2005-2011)**

The first period was characterized by high biomasses of krill and mesozooplankton in the southwestern and central BS, and high biomass of amphipods in the north (Fig. SM3-1). The southwest also had high biomass of advected 0-group fish, mainly driven by sporadically high occurrence of 0-group herring (2006, 2008), haddock (2009), cod (2011), and redfish (2007) (Eriksen et al. 2017). Sponges were more frequent in the south reflecting the suitable bank-habitat overflooded by warm Atlantic waters coming into the SW Barents Sea bringing in high biomasses of zooplanktonic food particles. High biomass of capelin and polar cod occurred in the north and northeast, likely due to their relatively large stocks.

During this period, the demersal fish community increased in biomass from a low level, mostly due to increase in the commercial species, causing an expansion in its distribution toward north and east) and giving high biomass of commercial demersal fish south of Svalbard and at Great Bank. The expansion was accompanied by a decline in small arctic fish.


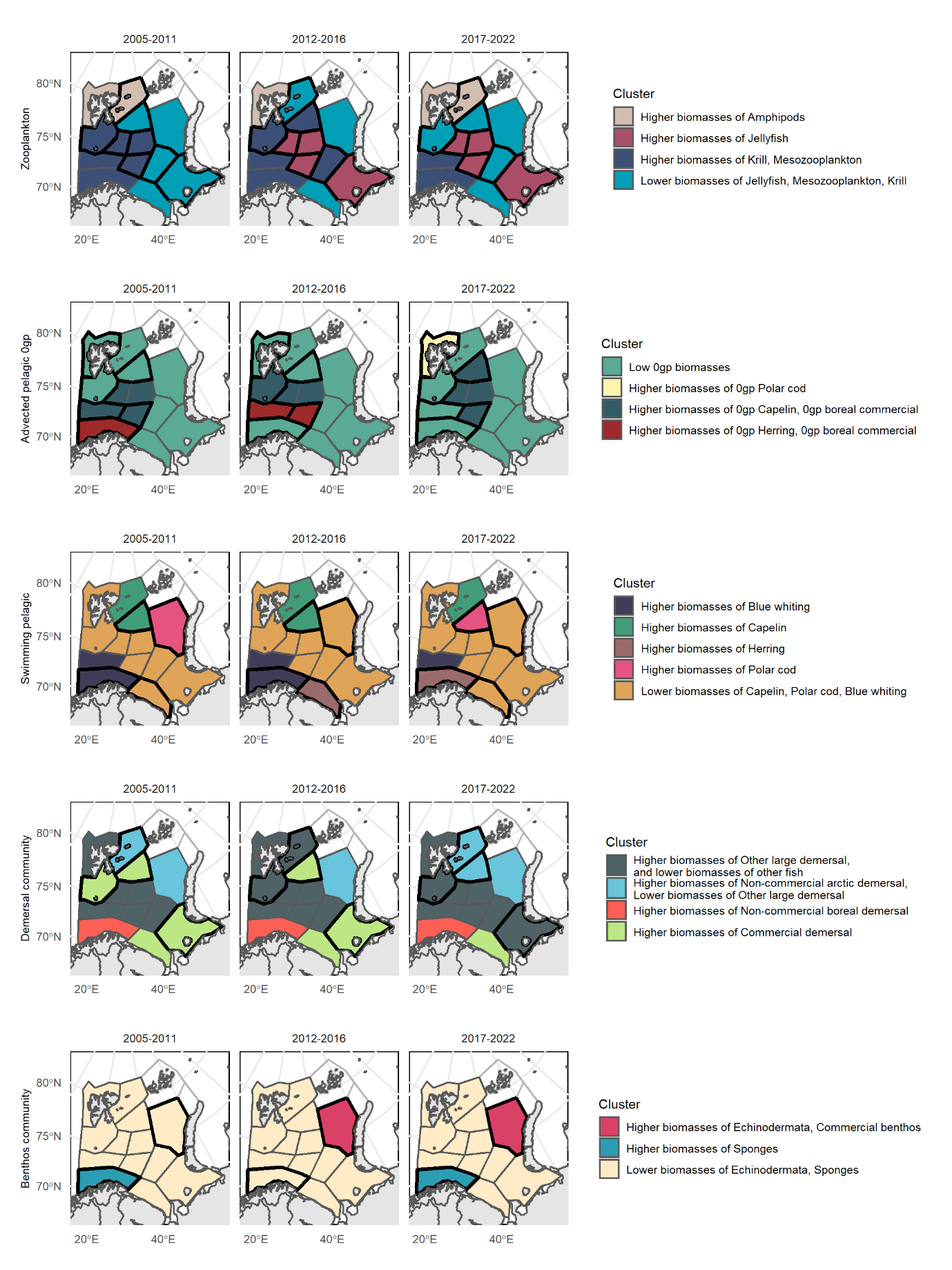


Fig S3-1. Space-time dynamics of the BS ecosystem components. Cluster analysis of average values of ecosystem components per polygon and period. Colors indicate the clusters for each group of ecosystem components. Bold boundaries highlight polygons that experienced a change in cluster type during the 18-year period.

### The peak-warming period (2012-2016)

This period was characterized by more jellyfish in the central and eastern BS, where mesozooplankton biomass became progressively less. Later in the period, the biomasses of mesozooplankton and krill around Svalbard increased. Occurrence of several strong year classes (cod in 2012-14, capelin in 2016, and herring in 2013) made the period exceptional. Boreal pelagic fish stocks occupied the southwest (blue whiting) and the southeast (young herring) and their biomasses were relatively high, while biomasses of cold waters species (capelin and polar cod) declined over the period. Demersal fish biomass levels were relatively high and stable during this period. Other large demersal fishes became more abundant to the south and east of Svalbard, likely increasing the predation on the small arctic fishes. Both the shrimp and the king crab biomasses reduced to lower average value, while the snow crab increased significantly in the northeast. The Echinodermata biomasses were also significantly higher in the northeast due to unusual high catches in 2012.

### The cooling period (2017-2022)

The BS became colder, and this period was characterized by high biomasses of amphipods in the north and jellyfish in the central and southeast, while generally low biomasses of krill, except eastern BS. Despite low biomass, more favourable cooler conditions after 2018 might explain occurrence of strong year classes of capelin (2019) and polar cod (2020, partly coming from the Kara Sea, WGIBAR 2021) and stocks redistribution to northwestern BS respectively. Polar cod juveniles and adults remained, however absent from their traditional area in the southeastern BS until the end of the time series. The biomass of demersal fish continued to decrease, mainly due to the decline of cod and haddock. Arctic species, among the demersal fish, did not recover to the higher biomass levels of the first period, except in the Franz Victoria Trough and Great Bank probably due to more suitable habitat and less predation from the declining cod stock. Snow crab show significantly high biomasses in the east. Echinodermata was still at its maximum in the northeast. Sponges' biomass was lower and below the long-term average in large part of the Barents Sea.
